# Molecular characterization of an MLL1 fusion and its role in chromosomal instability

**DOI:** 10.1002/1878-0261.12423

**Published:** 2018-12-31

**Authors:** Sreejit Parameswaran, Frederick S. Vizeacoumar, Kalpana Kalyanasundaram Bhanumathy, Fujun Qin, Md. Fahmid Islam, Behzad M. Toosi, Chelsea E. Cunningham, Darrell D. Mousseau, Maruti C. Uppalapati, Peter C. Stirling, Yuliang Wu, Keith Bonham, Andrew Freywald, Hui Li, Franco J. Vizeacoumar

**Affiliations:** ^1^ Department of Pathology and Laboratory Medicine, Cancer Cluster College of Medicine University of Saskatchewan Saskatoon Canada; ^2^ Department of Pathology School of Medicine University of Virginia Charlottesville VA USA; ^3^ Cell Signaling Laboratory Departments of Psychiatry and Physiology University of Saskatchewan Saskatoon Canada; ^4^ Terry Fox Laboratory British Columbia Cancer Agency Vancouver Canada; ^5^ Department of Biochemistry, Cancer Cluster College of Medicine University of Saskatchewan Saskatoon Canada; ^6^ Cancer Research Saskatchewan Cancer Agency Saskatoon Canada

**Keywords:** chromosomal instability, chromosomal rearrangement, fusion proteins, mitotic checkpoint, tumor heterogeneity

## Abstract

Chromosomal rearrangements involving the mixed‐lineage leukemia (*MLL1*) gene are common in a unique group of acute leukemias, with more than 100 fusion partners in this malignancy alone. However, do these fusions occur or have a role in solid tumors? We performed extensive network analyses of MLL1‐fusion partners in patient datasets, revealing that multiple MLL1‐fusion partners exhibited significant interactions with the androgen‐receptor signaling pathway. Further exploration of tumor sequence data from TCGA predicts the presence of MLL1 fusions with truncated SET domain in prostate tumors. To investigate the physiological relevance of MLL1 fusions in solid tumors, we engineered a truncated version of *MLL1* by fusing it with one of its known fusion partners, *ZC3H13*, to use as a model system. Functional characterization with cell‐based assays revealed that MLL1‐ZC3H13 fusion induced chromosomal instability, affected mitotic progression, and enhanced tumorsphere formation. The MLL1‐ZC3H13 chimera consistently increased the expression of a cancer stem cell marker (CD44); in addition, we detected potential collateral lethality between DOT1L and MLL1 fusions. Our work reveals that MLL1 fusions are likely prevalent in solid tumors and exhibit a potential pro‐tumorigenic role.

**Abbreviations**
7AAD: 7‐aminoactinomycin D
C7: clone 7
C9: clone 9
CIN: chromosomal instability
CNV: copy number variations
CSCs: cancer stem cells
DMEM: Dulbecco's modified Eagle's medium
DOT1L: disruptor of telomeric silencing 1‐like
DPBS: Dulbecco's PBS
EGFR: epidermal growth factor receptor
GAPDH: glyceraldehyde 3‐phosphate dehydrogenase
GTEx: genotype‐tissue expression
MLL‐1: mixed‐lineage leukemia
NEPC: neuroendocrine prostate cancer
RFP: red fluorescent protein
SET: Su(var)3‐9, enhancer‐of‐zeste and trithorax
shRNA: short hairpin RNA
TAE: Tris‐base‐Acetic acid‐EDTA
TCGA: The Cancer Genome Atlas
ZC3H13: zinc finger CCCH domain‐containing protein 13

## 1 Introduction

Chromosomal rearrangements often arise due to defects in the DNA damage repair pathway or DNA synthesis pathway (Hasty and Montagna, 2014). Recent developments in both genome and transcriptome sequencing have revealed numerous chromosomal rearrangements that may function as enabling factors for tumor maintenance and progression (Kumar *et al*., 2016). As fusion proteins are expressed exclusively in tumor cells and may contribute to tumor progression, they can act as targetable vulnerabilities. This makes molecular characterization of fusion proteins an intensively pursued area of research for therapeutics (Medves and Demoulin, 2012). For example, BCR‐ABL fusions that are found in multiple malignancies (Kumar‐Sinha *et al*., 2008; Salesse and Verfaillie, 2003) are being used as effective targets for cancer therapies. Fusion proteins that do not arise from chromosomal rearrangements but rather are derived from transcripts that are prematurely terminated, are also known to be involved in tumorigenesis (Kowarz *et al*., 2012). However, these gene fusions are not widely prevalent across cancers. For example, the relatively well‐studied EML‐ALK fusion in non‐small‐cell lung cancer (Morodomi *et al*., 2014) or the PML‐RAR fusion in acute myelogenous leukemia (Saeed *et al*., 2011) is found in only 2–5% or 6–10% of these malignancies, respectively. Therefore, it is often difficult to determine the vulnerable regions of the chromosomes and gauge the effectiveness of these chimeras as therapeutic targets.

One exception to this is the mixed‐lineage leukemia (*MLL*) gene, the locus of which is known to be a recombination hot spot (Zhang *et al*., 2012). *MLL1* is important for epigenetic maintenance of *Hox* gene expression and is required for normal fetal and adult hematopoiesis (Alharbi *et al*., 2013; Ballabio and Milne, 2012, 2014). Chromosomal rearrangements of the *MLL1* gene usually involve the fusion of the N‐terminal region of MLL1 with a variety of partners to create fusions that account for most cases of the MLL1‐associated leukemia (Li and Ernst, 2014; Marschalek, 2016; Slany, 2009; Winters and Bernt, 2017). So far, over 100 different MLL1‐fusion partners have been reported in acute leukemia (Marschalek, 2016; Meyer *et al*., 2009, 2013, 2018; Sanford *et al*., 1993; Tkachuk *et al*., 1992). In addition, *de novo* formations of MLL1‐fusion proteins have been reported in patients that undergo chemotherapy (Faller *et al*., 2009; Shapiro *et al*., 2007; Xie *et al*., 2013). Although patients with MLL1 fusions in leukemia are highly prone to relapse and may require the early‐phase intensification of treatment, the exact role of MLL1 fusions in other malignancies remains unclear (Tomizawa *et al*., 2007). To address this, we explored the interaction landscape of MLL1‐fusion partners and predict the prevalence of these fusions in solid tumors. We also engineered a model system to evaluate the relevance of MLL1 fusions in solid tumors. Our work revealed that MLL1 fusions potentially play an important role in solid tumors.

## 2 Materials and methods

### 2.1 Construction of MLL1‐ZC3H13 fusion plasmid

We designed our MLL1‐ZC3H13 fusion construct based on previously described MLL1‐ZC3H13 gene fusion (Duhoux *et al*., 2012). Briefly, the previously identified head‐to‐head gene fusion of *MLL1* exon 9 and *ZC3H13* intron 1 reverse sequence in a clinical case was codon optimized and synthesized (Genescript, Piscataway, NJ, USA) to insert into the pUC plasmid vector. BP cloning (part of Gateway recombination cloning technology; Thermo Fisher Scientific, Waltham, MA, USA) was performed using the pUC plasmid vector and pDONR221 to obtain the Gateway entry clone with the construct. LR cloning (part of Gateway recombination cloning technology; Thermo Fisher Scientific) was performed using pDONR221 with MLL1‐ZC3H13 fusion (Gateway entry clone with construct) and pLX304 (Destination vector) for 1 h at room temperature. The recombined destination vector with fusion construct was transformed in *Escherichia coli* One Shot ccdB Survival 2 T1R Chemically Competent Cells (Thermo Fisher Scientific) according to the manufacturer's protocol. Isolated colonies were sequenced to confirm the recombined plasmid and, once a suitable candidate was identified, to generate sufficient quantities of the plasmid DNA for further use. Similarly, for localization studies, the MLL1‐ZC3H13 fusion construct in pDONR221 was cloned into pLK0.1 plasmid with C‐term EGFP tag.

### 2.2 Cell culture, transfections, and transductions

HCT116 colon cancer cell line (ATCC, Manassas, VA, USA) was cultured in the suggested McCoy5A media. Transfections were carried out using X‐tremeGENE 9 DNA Transfection Reagent (Roche Life Science, Basel, Switzerland) as per the manufacturer's instructions. Pooled lentivirus containing the MLL1‐ZC3H13 fusion construct was prepared by transfecting HEK293T cells. Briefly, the cells were transfected with psPAX2, pMD2.G, and pLX304 with MLL1‐ZC3H13 fusion construct simultaneously; after 18 h, media were replaced with Dulbecco's modified Eagle's medium (DMEM) containing 2% (w/v) BSA. The lentivirus was collected after 24 and 48 h, and pooled and stored at −80 °C. Transducing HCT116 cells with pooled lentiviruses containing MLL1‐ZC3H13 fusion construct generated stable MLL1‐ZC3H13 fusion clones. Transduced cells were selected with Blasticidin (initial 1 and later 5 μg·mL^−1^) for 10 days (pLX304 with V5 tag – Destination vector). The clones were selected once there was complete cell death in a parallel control plate (i.e. no viral infection) and then expanded before assays. Similarly, pLX304 vector control lentivirus was prepared, transduced into HCT116 parental cells, and selected. Lipofectamine‐based transfection was also performed as per the manufacturer's recommendation on HCT116 cells with pLK0.1 plasmid containing MLL1‐ZC3H13 fusion construct with GFP tag, 24 h prior to microscopy.

### 2.3 Clone validation

The isolated clones (V5‐tagged) were tested for the expression of MLL1‐ZC3H13 fusion construct by flow cytometry using the anti‐V5 antibody. Along with clones, a parental control and vector control were also used for the validation assays. Briefly, for flow cytometry, the single‐cell population of clones and controls were fixed and permeabilized using reagents from the kit following the manufacturer's suggestion (BD Cytofix/Cytoperm, BD Biosciences, Mississauga, Canada; 554714). The cells were stained with anti‐V5 antibody (Abcam, Toronto, Canada, AB9116) and then with secondary goat anti‐rabbit IgG antibody conjugated with AF488 (Thermo Fisher Scientific, A11008). The data were subsequently analyzed by flowjo ^®^ software (FlowJo LLC., Ashland, OR, USA, version 9.9 for Mac). The stemness of the clones was also assessed by flow cytometry following direct staining of cells without fixation or permeabilization using FITC‐conjugated mouse anti‐Human CD44 (BD Biosciences, 555478) along with Isotype control (FITC‐conjugated Mouse IgG2b Κ‐BD Biosciences, 555742).

### 2.4 RT‐PCR

RNA was isolated from cell pellets using RNeasy mini kit (Qiagen, Hilden, Germany, 74104) according to the manufacturer's instructions including DNase treatment (Qiagen, 79254). RNA quantification was performed using a NanoDrop 2000c spectrophotometer (Thermo Fisher Scientific) and RNA integrity was verified spectrophotometrically by A260/A280 ratios between 1.8 and 2.0 and A260/A230 ratios > 2. Equal quantities of RNA were used to generate cDNA using the High capacity cDNA Reverse transcription kit (Thermo Fisher Scientific) according to the manufacturer's instructions. Primers specific to the MLL1‐ZC3H13 insert junction (Fp – CCCTCAGAGCCAAAGAAGAA; Rp – ACAAGAAAGCTGGGTTCTGA) were used for this study. PCR was performed using PowerUp™ SYBR™ Green Master mix for 40 cycles. The amplified PCR products (154 bp) were then run on 1% agarose gel in Tris base‐Acetic acid‐EDTA (TAE) buffer along with SYBR™ Safe for 45 min at 100 V prior to visualization along with Generuler 1 kb DNA ladder (Thermo Scientific). Appropriate controls including blanks were also run along with the samples (Data not shown).

### 2.5 Flow cytometry

Cell cycle analysis and phospho‐histone H3 staining for mitotic cell detection are discussed briefly in the mitotic defect assays. Besides these assays, live‐dead analysis and CD44 staining were also performed in this study.

Live‐dead assay was carried out by staining with 7‐aminoactinomycin D (7AAD). Test cells and appropriate control cells were trypsinized and neutralized with the same media in which they were cultured and then washed once with Dulbecco's PBS (DPBS) prior to adding 5 μL 7AAD (BD Biosciences, 559925) in 500 μL DPBS per million cells. The cells were incubated at room temperature in the dark for 10 min and washed twice with DPBS, and run through a Beckman Coulter CytoFLEX flow cytometer (Beckman Coulter Life Sciences, Indianapolis, IN, USA) at 488 nm and analyzed using cytexpert V2.1 software (Beckman Coulter Life Sciences).

CD44 staining was performed on cells which were harvested and washed three times with ice‐cold PBS containing 0.25% FBS. Cells were incubated with FITC‐conjugated mouse‐anti‐human CD44 antibody (BD, 555478) or FITC‐conjugated mouse IgG2b antibody (BD, 555742) as isotype control, for 30 min at 4 °C in the dark. Cells were then washed thrice with PBS, run through a Beckman Coulter CytoFLEX flow cytometer at 488 nm, and analyzed with cytexpert V2.1 software.

### 2.6 Microscopy

Confocal microscopy was used to examine the subcellular localization of MLL1‐ZC3H13 fusion construct. Cells of selected clones (pLK0.1 construct with GFP tag and MLL1‐ZC3H13 fusion) and controls (parental HCT116 and vector control) were grown in 96‐well plates with a glass bottom (Thermo Fisher Scientific, 164588). Hoechst 33342 live cell nuclear staining was performed as previously reported (Chazotte, 2011) and the cells were then maintained in FluoroBrite DMEM Media (Thermo Fisher Scientific – A18967‐01). Localization of the expressed MLL1‐ZC3H13 fusion construct to the nucleus was confirmed using an FV300 confocal laser scanning biological microscope (Olympus, Tokyo, Japan) in an incubator chamber with heated stage. Cells of selected clones (pLX304 construct with V5 tag and MLL1‐ZC3H13 fusion) and controls (parental HCT116 and vector control) were grown in 96‐well plates with a glass bottom, fixed, and permeabilized. The cells were stained with Hoechst 33342 live cell nuclear stain, and then observed under a confocal microscope for mitotic defects and nuclear abnormalities. The images acquired were analyzed for nuclear size, mitotic defects, and protein localization studies with metaxpress software (Molecular Devices LLC., San Jose, CA, USA).

### 2.7 Mitotic defect assays

To confirm the defects in mitosis are due to the expression of MLL1‐ZC3H13 fusion protein, cell cycle analysis and spindle assembly checkpoint activation were assayed. Cell cycle analysis was performed by flow cytometry on cells of selected clones (pLX304 construct with V5 tag and MLL1‐ZC3H13 fusion) and vector control. Following plating of fresh stocks, cells were grown for increasing lengths of time (24, 48, 72, and 96 h), following which they were trypsinized, fixed with pre‐chilled 70% ethanol, and stored at −20 °C for 2 h or more. The cells were washed with DPBS and stained with propidium iodide (10 μg·mL^−1^) containing RNase A (5 μg·mL^−1^) final concentration. The data were then analyzed using flowjo software. Spindle checkpoint activation defect was assayed following synchronization as described previously (van de Weerdt *et al*., 2005). In brief, cells of selected clones (pLX304 construct with V5 tag and MLL1‐ZC3H13 fusion) and vector control were blocked with thymidine (2.5 mm) for 24 h twice to induce synchronization, and then released with nocodazole (0.3 μm). For analysis with phospho‐specific antibodies, cells were released from thymidine block for 15 h in the presence of nocodazole. Mitotic cells were collected by shake off and harvested after release with nocodazole at 0, 9, 12, and 15 h as described previously (van de Weerdt *et al*., 2005). Cells were washed and stained with AF488 conjugated rabbit phospho‐histone H3 antibody (Cell Signaling Technology, Danvers, MA, USA, 9780S). Flow cytometry was performed and the data were then analyzed using flowjo software.

### 2.8 Proliferation and doubling time

To understand the effect of MLL1‐ZC3H13 fusion construct expression in cells, proliferation and doubling rate of the selected clones (pLX304 construct with V5 tag and MLL1‐ZC3H13 fusion) and controls (Vector control) were determined. The cells were grown in 12‐well plates at varying dilutions (25 000, 50 000, and 100 000 cells/well) for 4 days. After every 24 h post‐inoculation, the cells were trypsinized and counted by trypan blue staining. The count values were used to calculate the doubling time as per the formula given below (Roth V. 2006 Doubling Time Computing, Available from: http://www.doubling-time.com/compute.php).

$$\begin{array}{cc} & \text{Doubling}\;\text{time}=\\ & \frac{\text{Time}\;\text{duration}\times \log(2)}{\log(\text{Final}\;\text{concentration})-\log(\text{Initial}\;\text{concentration})}. \end{array}$$

### 2.9 *In vitro* wound healing assay

The *in vitro* scratch, or wound healing assay, an easy method to study cell migration, was performed as previously described with minor modifications (Liang *et al*., 2007). Once cells reached 80% confluence in 6‐well plates, they were starved for growth factors for 7 h. Briefly, scratches were generated with a P200 tip, and static images were taken of three fixed spots along each scratch at 0 h (right after scratch), 24, 48, and 72 h after scratching. Static images were recorded on an EVOS™ XL Core Cell Imaging System (Thermo Fisher Scientific), and distances were measured subsequently from the images.

### 2.10 Soft agar assay

Anchorage‐independent proliferation was examined in selected clones (pLX304 construct with V5 tag and MLL1‐ZC3H13 fusion) and vector control. For this assay, cells were seeded in 6‐well plates (1 × 10^5^ cells/well). Plates were prepared by making a bottom agar layer of 2.5 mL warm agar mix (43 °C) containing solidified 2× McCoy5A, 20% FBS, and 0.8% agarose to each well. On solidifying, 2 mL of top agar mix (2× McCoy5A, 20% FBS, and 0.7% agarose) containing cells was layered over the bottom layer. The cells were incubated in humidified 5% CO_2_ and 95% air at 37 °C and fed by adding 0.5 mL of normal media containing 10% FBS every 7 days. Twenty‐eight days after seeding, colonies were counted at 10× magnification (EVOS™ XL Core Cell Imaging System) and the data were analyzed using metaxpress software.

### 2.11 Tumorsphere forming assay

The 3D‐tumorsphere‐culture model represents *in vivo* conditions for the spontaneous aggregation of cancer cells in spheroids. On attaining 60% confluency, single cell suspension of selected clones (pLX304 construct with V5 tag and MLL1‐ZC3H13 fusion) and vector control were counted and seeded into half of a 96‐well plate/clone (2000 cells per 100 μL per well) on ultra‐low adhesion plates (Corning Incorporated, Corning, NY, USA, 3474) in tumorsphere medium (Stem Cell Technologies, Vancouver, Canada). Following incubation at 37 °C for 1 week, several wells were photographed prior to collection for counting. For counting, two tubes of 24 wells were collected for each cell line. Cells were pelleted and medium‐aspirated, and 300 μL trypsin was added to each tube. Cell suspension was pipetted intermittently until a single cell suspension was achieved and 300 μL of medium containing FBS was added. Suspension was mixed and total cells were counted using a hemocytometer, and data were analyzed.

### 2.12 Drug inhibition assay

Pinometostat (EPZ5676; Selleckchem, Houston, TX, USA, S7062) in DMSO was diluted in media to final concentrations of 10 nm, 100 nm, 1 μm, and 10 μm. The selected clones (pLX304 construct with V5 tag and MLL1‐ZC3H13 fusion) and vector and parental control were plated at 1000 cells/well in 96‐well plates along with media containing different concentrations of drug and 0.1% DMSO as control. The plates were incubated with drug for 3 days and resazurin assay was performed subsequently for cell viability and read in a spectrophotometer (Molecular Devices SpectraMax M5, Molecular Devices LLC., San Jose, CA, USA). Another group of 6‐well plates (initial seeding of 0.3 × 10^6^ cell/well) treated with drug along with respective control was maintained for 15 days. On every third day, the cells were trypsinized, counted, and reseeded into new 6‐well (for drug treatment maintenance) and 96‐well plates (for resazurin assay after third day) along with media containing the respective drug concentration. The remaining cells were used for cell cycle analysis using flow cytometry as per the protocol described earlier (Daigle *et al*., 2013).

### 2.13 Silencing of gene expression using **short hairpin RNA (shRNA**)

To silence specific gene using shRNA, cells (C7, C9, and Vector control) were transduced with lentivirus containing shRNA sequences specific to DOT1L. Three shRNA sequences for DOT1L were transduced separately. A shRNA sequence specific to red fluorescent protein (RFP; Sigma‐Aldrich, St. Louis, MO, USA) was used as a non‐targeting control. For each transduction, 0.5 mL of each shRNA lentivirus was added to 2 × 10^5^ cells in a 35‐mm dish in a final volume of 3 mL with 8 μg·mL^−1^ of polybrene (Sigma‐Aldrich, 107689). Twenty‐four hours after transduction, the media were removed and replaced with media containing 2 μg·mL^−1^ puromycin (Fisher Scientific, Ottawa, Canada, BP2956100) for selection. Cells were selected for a minimum of 48 h prior to use in experiments.

Proliferation assay was performed by growing puromycin‐selected cells in an automated live‐cell analyzer (incucyte®s3 – Essen BioScience Inc., Ann Arbor, MI, USA). Puromycin‐selected cells were seeded onto 12‐well plates (~ 50 000/well) and incubated for 5 days. Images were taken every second hour and the area of growth was computed using incucyte® s3 Software (Essen BioScience Inc.). The values were plotted as a graph after calculating the fold level change in proliferation.

qRT‐PCR as validation for shRNA silencing was performed using RNA extracted from cell pellets of RFP‐ and DOT1L‐transduced C7 and C9 clones and vector control, 48 h after initial puromycin selection using RNeasy mini kit (Qiagen, 74104) according to the manufacturer's instructions including DNase treatment (Qiagen, 79254). RNA quantification was performed using a NanoDrop 2000c spectrophotometer (Thermo Fisher Scientific) and RNA integrity was verified spectrophotometrically by A260/A280 ratios between 1.8 and 2.0 and A260/A230 ratios > 2. Equal quantities of RNA were used to generate cDNA using the High capacity cDNA Reverse transcription kit (Thermo Fisher Scientific) according to the manufacturer's instructions. *DOT1L* expression levels were evaluated by real‐time PCR gene expression assay using PowerUp™ SYBR™ Green Master mix according to manufacturer's instructions (Fp – ACAGGGTTGATGGCAGAGAC; Rp – TGACACGCATATGACCCAGT). The change in gene expression was analyzed using the ΔΔCt method. Glyceraldehyde 3‐phosphate dehydrogenase (GAPDH) was used as an internal control.

### 2.14 Computational analyses

Gene expression analysis was carried out to compare the expression of a query gene with a normal counterpart across 24 different cancers using data from The Cancer Genome Atlas (TCGA) and T‐ALL data from European Bioinformatics Institute (EMBL‐EBI). Gene expression data from TCGA were also used to compute the correlation between the fusion partners and MLL1. The open access interaction repository BioGRID (Version 3.4.150) was used to download all the interactions of the fusion partners to build the interaction network. Data from cBioPortal were used to compute the gene amplification, deletion, and mutation status for the fusion partners in multiple cancers. The open source software platform Cytoscape (Version 3.6.0) was used to integrate the data from BioGRID to construct the final visualization of the interactions. RNA‐seq data, including 117 normal prostate tissues from GTEx (genotype‐tissue expression), 50 solid normal prostate tissues, and 502 primary solid tumor from TCGA, were analyzed to identify fusion RNA by the EricScript framework/library open software package as described previously (Benelli *et al*., 2012).

### 2.15 Complementation assay

Temperature‐sensitive *pds1‐128* mutant yeast strain was transformed with MoBY‐ORF plasmids encoding *PDS1* as a positive control, an irrelevant gene *CAN1* as a negative control or *URA3* marked plasmids expressing human ZC3H13 isoforms (Ho *et al*., 2009). Yeast growth was measured by liquid growth curve by monitoring A_600_ nm over 24 h at 34 °C in a TECAN M200 plate reader (Tecan Group Ltd., Mannedorf, Switzerland).

### 2.16 Statistical analysis

The statistical significance of every experiment was calculated from three separate biological experiments. The corresponding *P* values calculated using Student's *t* test are indicated in the main figures. The calculated *P* values were represented as ‘*’,’**’ and ‘***’ for significance < 0.05, < 0.001, and < 0.0001, respectively, in Figures 2, 3, 4, 5, and S4–S6).

## 3 Results

### 3.1 Network and expression analyses predict the involvement of MLL1‐fusion partners in solid tumors

**Figure 1:**
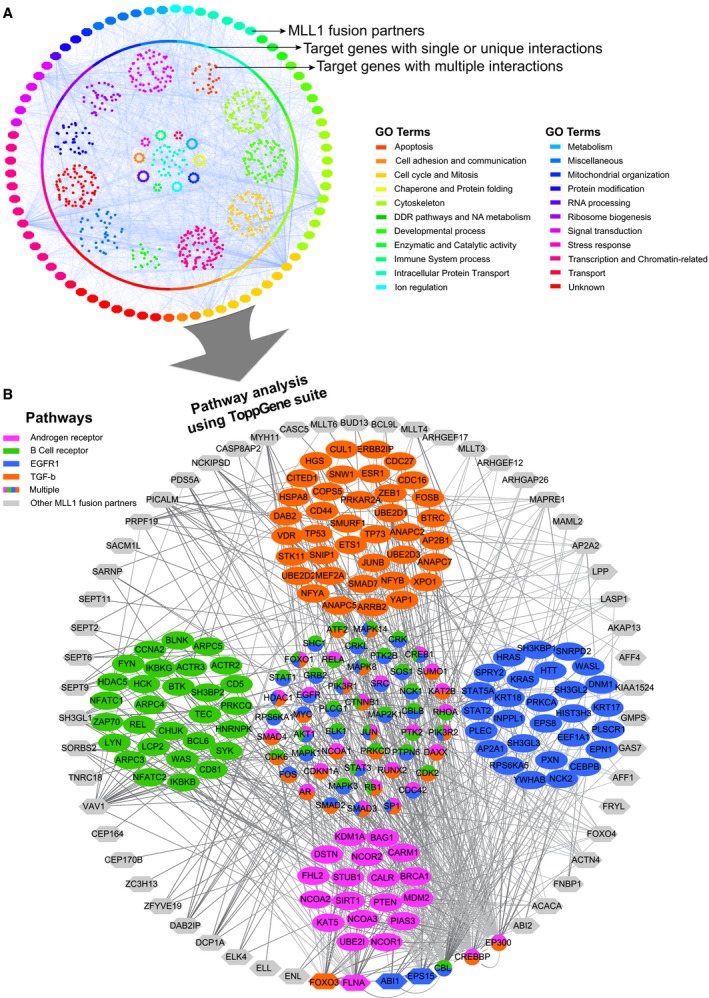
Interactions of MLL1‐fusion partners. Network of interactions of the fusion partners of MLL1 as derived from BioGRID. (A) The outer ring of nodes represents all the MLL‐1 fusion partners and the inner circle of nodes represents unique interactions. The inner core represents genes that interact with multiple fusion partners. Color coding is done based on a slim version of GO biological processes. (B) Represents a zoomed‐in version of the genes that have multiple interactions with four enriched pathways (see text for *P* value significance). The outer ring of gray nodes represents the MLL1‐fusion partner genes and the inner group of nodes represents the target interacting genes grouped according to the pathways relevant in cancer. Genes that belong to multiple pathways are arranged in the core of the network. The program Cytoscape was used to generate the interaction network and ToppGene suite was used for enrichment analyses.

Very little is known about the prevalence of MLL1 fusions in solid tumors and how chromosomal rearrangements of *MLL1* can benefit cancer cells by partnering with specific fusion proteins. To elucidate this, we analyzed the expression pattern of some of the common fusion partners of MLL1 and found these to be down‐regulated in solid cancers. For example, *AFF1* and *MLLT3* are down‐regulated in bladder, breast, and lung tumors compared with their expression in corresponding normal tissues (*P* < 0.001; Fig. S1). We next asked whether this down‐regulation of the fusion partners correlated with *MLL1* expression across multiple cancers. A correlation clustergram was plotted for all the reported MLL1‐fusion partners across 18 tumor types. Interestingly, expression of most of the fusion partners including the high‐frequency partners like *AFF1, MLLT1, MLLT3, MLLT10, ELL*, and *MLLT4*, were positively correlated with MLL1 expression irrespective of the tumor type (Fig. S2). Thus, it would appear that MLL1‐fusion partners are linked to the expression of *MLL1* not only in hematopoietic tumors but also in solid tumors. To investigate this link further, we explored the interaction landscape of the MLL1‐fusion genes. We curated functional interactions of 75 of the 82 well‐established fusion partners for which interactions are available in BioGRID and asked in which biological pathway they are specifically enriched. The resulting collated network is unexpectedly dense and highly interconnected with 2306 nodes and 3805 edges, although many outstanding nodes are also apparent (Fig. 1A). In total, we found the 75 fusion genes interacted with over 2305 molecules that are involved in multiple biological processes (Fig. 1A). Although 1462 interactions were exclusively found to be specific for each fusion partner, 52 fusion genes exhibited a high degree of connectivity with 767 proteins (Fig. 1B). These common interactions are enriched for proteins that are involved in transcriptional regulation and chromatin remodeling (Fig. S3). Specifically, pathway analysis using the ToppGene suite (Chen *et al*., 2009) found that the interactions of the MLL1‐fusion partners were primarily with proteins involved in androgen‐receptor signaling pathway (Bonferroni‐corrected *P* value, 3.019E‐31), TGF‐beta receptor signaling pathway (Bonferroni‐corrected *P* value, 3.080E‐38), B‐cell receptor signaling pathway (Bonferroni‐corrected *P* value, 6.777E‐34), and Epidermal growth factor receptor 1 (EGFR1) signaling pathway (Bonferroni‐corrected *P* value, 1.385E‐31; Fig. 1B, Table S1). In fact, some of the MLL1‐fusion partners themselves are involved in these pathways. For example, FOXO3 is involved in the TGF‐beta receptor signaling pathway and EPS15 is involved in the EGFR1 signaling pathway (Massague and Gomis, 2006; Tomas *et al*., 2014). While interactions with the components of B‐cell receptor signaling or TGF‐beta receptor signaling pathways were not surprising, given the prevalence of MLL1 fusions in leukemia (Buchner and Muschen, 2014; Rouce *et al*., 2016), the observation of extensive interactions with components of the EGFR and androgen signaling pathways was intriguing, as these pathways are primarily dysregulated in solid tumors. This is important as, to our knowledge, there has only been one report on the incidence of MLL1 fusions in solid tumors, and this was reported in prostate cancer (Chowdry *et al*., 2016) and, until now, most of the information regarding MLL1 translocations has been derived from leukemia patients.

We also examined cBioPortal data for all the available fusion genes for their roles in cancer. Of the 78 fusion genes, we found that 33 are mutated, 62 are amplified, and 15 are deleted in at least any five cancers listed in the database (Fig. S4). Of note, we found most of the fusion partners were altered in neuroendocrine prostate cancer (NEPC) (Fig. S4). Since MLL1‐fusion proteins are highly altered in NEPC and many of the fusion partners had interactions with the androgen‐receptor signaling pathway, we next set out comprehensively to explore MLL1 fusions in prostate tumors.

### 3.2 Predicted MLL1 fusions in prostate tumors affect the C‐terminal SET domain containing the H3K4 methyltransferase activity

**Figure 2:**
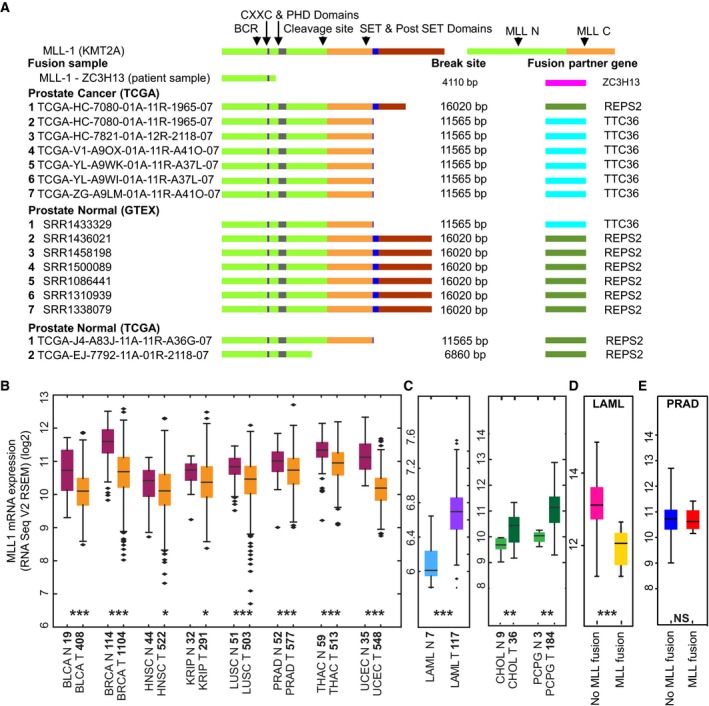
Search for MLL1‐fusion and its clinical relevance in solid tumors. (A) Schematic of wild‐type MLL1 (*KMT2A*) and MLL1‐fusion with other genes (fusion partners) in patient samples available from TCGA, GTEx‐Prostrate and TCGA normal datasets. Major functional domains and the proteolytic cleavage site of wild‐type MLL1 are indicated along with the break site on fused MLL1 due to fusion partner junction. (B) Relative expression of MLL1 RSEM normalized mRNA in samples of various cancers (abbreviation of each cancer is described in the TCGA portal) from the TCGA database showing low expression of *MLL1* in tumor samples than in normal samples; *P* values (Mann–Whitney *U* test) indicated by *P < 0.05, **P < 0.001, ***P < 0.0001; *n*‐values of each sample provided on the *x*‐axis. (C) Relative expression of MLL1 RSEM normalized mRNA in samples of LAML, CHOL, and PCPG, compared with their normal counterparts. (D) Relative expression of MLL1 RSEM normalized mRNA in LAML samples with and without MLL1‐fusion from the TCGA database. (E) Relative expression of MLL1 RSEM normalized mRNA in Prostate adenocarcinoma (PRAD) samples with and without MLL1‐fusion from the TCGA database (NS denotes not significant).

As mentioned above, the high alteration frequency of MLL1‐fusion partners in NEPC and their interactions with androgen‐receptor signaling pathway components (Figs 1 and S4), which are important in prostate cancer progression (Mazaris and Tsiotras, 2013; Ramsay and Leung, 2009), prompted us to explore the tumor sequencing data from TCGA to identify gene fusions of MLL1 in prostate cancer. Along these lines, there were two cases of MLL1 fusions reported in prostate cancer (Chowdry *et al*., 2016) and so we decided to use the RNA‐seq data derived from prostate cancer patients in the TCGA database to investigate the occurrence of MLL1 fusions. Altogether, 502 primary prostate tumors from TCGA along with several controls from normal tissues derived from both TCGA and GTEx were analyzed for the presence of MLL1 fusions. There were data on 50 normal prostate tissues from TCGA and 117 normal prostate samples from GTEx. The gene‐fusion analysis was performed by EricScript as described previously (Benelli *et al*., 2012). We identified seven prostate cancer patients from the TCGA data who exhibited fusion events (Fig. 2A). Interestingly, we also found seven samples from the normal GTEx prostate samples and two normal samples from TCGA that also showed fusion events. However, six of the seven prostate cancer patients have lost the C‐terminal SET domain containing the H3K4 methyltransferase activity. In contrast, of the seven cases from the GTEx dataset, in only two cases was the SET domain affected, while the remaining five had an intact SET domain (Fig. 2A). In addition, our work indicated the presence of additional novel fusion partners such as *TTC36* in prostate tumors, located in the same 11q23.3 locus and separated by < 640 bp in a normal chromosome 11q23.3 locus. Interestingly, like ZC3H13 and most other fusion partners, *TTC36* is also lost in other solid tumors such as bladder and breast (Fig. S4). Overall, our analyses predicted that incidences of MLL1 fusions with truncated SET domain are prevalent in prostate tumors, albeit with low frequency, and single cell sequencing analyses may reveal the magnitude of such alterations.

### 3.3 Expression of the *MLL1* gene may not be a reliable biomarker to detect the incidence of MLL1‐rearrangement in solid tumors

Recent studies in leukemia demonstrated that monitoring the expression of the *MLL1* gene may help in the identification of patients with MLL1 rearrangement, as the expression of *MLL1* is lost when there is a fusion (Cerveira *et al*., 2009; Chen *et al*., 2008; Liu *et al*., 2014). However, *MLL1* gene has been reported to be down‐regulated in breast, head, and neck cancers (Figueiredo *et al*., 2015; Rabello Ddo *et al*., 2013), and it is not clear whether this would be of any diagnostic value in solid tumors. We took advantage of the TCGA database and investigated the expression of the *MLL1* gene across various cancers for which matched expression data in normal and cancer tissues are available. Interestingly, we found that expression of *MLL1* is reduced in most solid tumors including bladder, breast, prostate, lung, and thyroid cancers compared with the expression in corresponding normal tissues (*P* < 0.001) (Fig. 2B). In addition, the copy number variations (CNVs) for *MLL1* at the chromosomal region, 11q 23.3, from solid tumors including breast, skin, and liver cancer patients showed both homozygous (more frequently) and heterozygous (much rarer) deletions (Fig. S5). This observation contrasts with the changes observed in CNV of leukemia patients who consistently exhibited amplifications of the chromosomal region, 11q 23.3 (Fig. S5). Similarly, in liquid (LAML) or solid‐like liquid tumors such as cholangiocarcinoma (CHOL) or pheochromocytoma and paraganglioma (PCPG), *MLL1* expression is elevated compared with normal healthy samples (Fig. 2C).

To test whether *MLL1* gene expression can be used as a biomarker to detect the incidence of MLL1‐rearrangement in solid tumors, we compared the expression of *MLL1* gene in leukemia patients that harbor MLL1‐fusion with the expression in those who do not harbor this fusion (Fig. 2D). Unlike liquid tumors, we did not find any difference in the expression of *MLL1* gene in prostate cancer patients with MLL1 fusions and other prostate cancer patients without MLL1‐fusion (Fig. 2E). Thus, monitoring the expression of *MLL1* gene alone might not add value in the identification of patients with MLL1 rearrangement in solid tumors. Since our observations indicate that MLL1 fusions are likely to have an important role in solid tumor biology, employing single cell tumor‐sequencing strategy to detect these chimeras will provide effective therapeutic approaches.

### 3.4 Modeling and functional characterization of MLL1‐recombination in cell‐based assays

**Figure 3:**
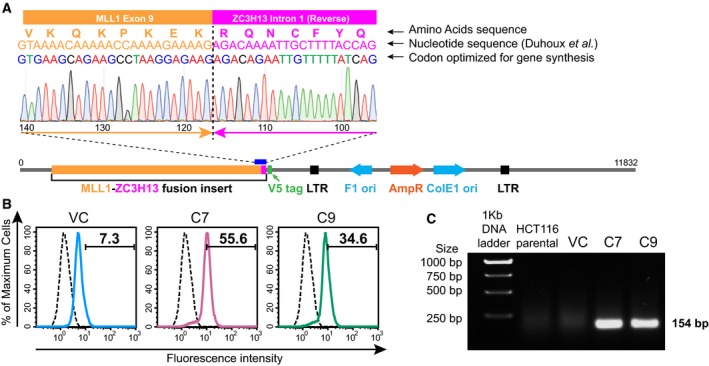
Validation of generated clones. (A) Chromatogram showing the junction of the fusion between MLL1 and ZC3H13 along with a schematic of the construct with codon optimization for efficient gene synthesis. Codon optimization done for gene synthesis without affecting the translated protein is indicated for comparison with previously reported fusion (Duhoux *et al*., 2012). (B) Flow cytometric analysis of HCT116 clones containing MLL1‐ZC3H13 fusion insert with V5 tag (HCT116 C7 – Pink and HCT116 C9 – Green inlaid with isotype control – black) and VC (vector control) – Blue (inlaid with isotype control – Black) following staining with antibody against V5 tag. Representative images show mean percentage values of three experiments. (C) Agarose gel showing RT‐PCR product (154 bp size) of fusion expression constructs in HCT116 clones containing MLL1‐ZC3H13 fusion insert compared with HCT116 parental and VC.

A fusion between *MLL1* and *ZC3H13* was found in cancer cells (Duhoux *et al*., 2012); this fusion also showed the loss of the SET domain as we observed in our TCGA analyses (Fig. 2A). *ZC3H13* was identified as a key driver of chromosomal instability (CIN) (Wang *et al*., 2004) and our previous work showed that *ZC3H13* might play an important role in spindle assembly checkpoint (Vizeacoumar *et al*., 2013). Using a lentivirus‐based system, we engineered a MLL1‐ZC3H13 chimera and expressed it as a stable cell line model for our studies. We chose to do these experiments in HCT116 cells as they have stable karyotype with intact DNA damage checkpoint and spindle assembly checkpoint functions (Waldman *et al*., 1995, 1996) and are commonly used as a model to study CIN (Amato *et al*., 2009; Lentini *et al*., 2007; McManus *et al*., 2009; Ryan *et al*., 2012; Schvartzman *et al*., 2010, 2011; Tomonaga *et al*., 2005; Waldman *et al*., 1996). The lack of an established karyotypically stable prostate cell line, which would have been ideal for studying CIN, made us choose HCT116 cells as a model system. We generated stable clones of the HCT116 origin that expressed the V5‐tagged MLL1‐ZC3H13 fusion, which was codon optimized for efficient gene synthesis (Fig. 3A). Expression of the fusion constructs was determined using the flow cytometry approach to detect V5 tag and was also validated using RT‐PCR (Figs 3B,C and S6A). Based on the level of V5 expression, two clones, C7 and C9, were expanded and used for the further studies.

**Figure 4:**
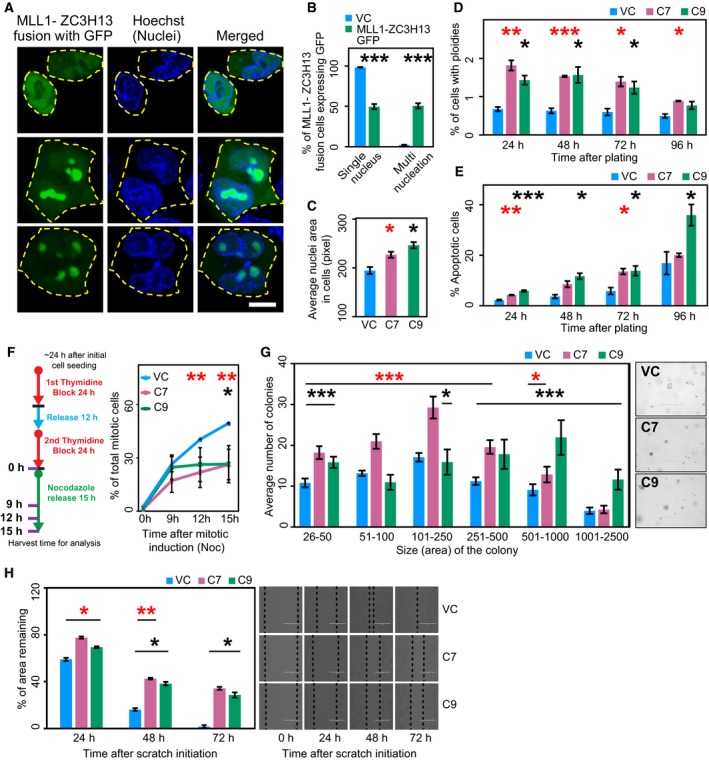
Phenotypic characterization of clones expressing MLL1‐ZC3H13 fusion. (A) Confocal microscopy of liposomal based transfection of HCT116 cells with plasmid vectors containing MLL1‐ZC3H13 fusion insert with GFP. The cells were photographed and the images were analyzed using metaxpress. Representative images of different types of localization patterns with blue nuclei (Hoechst‐stained) and the MLL1‐ZC3H13 fusion on expression show GFP (Green) inside the nuclear membrane with occasional cytoplasmic diffusion. Scale bar: 10 μm. (B) Enumeration of different types of nucleation (as percentage values) in cells expressing MLL1‐ZC3H13 fusion protein with GFP tag. (C) Average nuclei size of Hoechst‐stained HCT116 cells containing C7, C9, and VC (vector control). (D) Determination of aneuploidy by flow cytometric analysis using DNA profiles at various time intervals in C7, C9, and vector control cells. Representative figures for cell cycle analyses are provided in Fig. S6. Histogram shows mean values of three experiments and calculated standard error bars. (E) Determination of apoptosis by flow cytometric analysis using DNA profiles at various time intervals in C7, C9, and VC cells. Histogram shows mean values of three experiments and calculated standard error bars. (F) Schematic representation of protocol used for double thymidine block and nocodazole release to assess mitotic defects. Line graph showing percentage of mitotic cells in C7, C9, and VC cells, as determined by phospho‐histone H3 staining using flow cytometry. Mean values of three experiments were used for the graph and standard error was also calculated. *P < 0.05, **P < 0.001, 2‐tailed Student's *t* test (Red * – HCT116 C7 and Black * – HCT116 C9). (G) Soft agar colony‐forming assay was performed by seeding equal numbers of C7, C9, and VC cells. Numbers and sizes of colonies were quantified after 4 weeks using images and analyzed with metaxpress. Representative images of colonies in soft agar shown on the side. (H) Wound healing scratch assay was made on 24‐h serum‐starved, confluent cells of C7, C9, and vector control. Cell migration quantification was assessed by calculating the area remaining in the central gap (delineated with dotted black line). Representative phase‐contrast microscopy pictures show the extent of closure in the scratch area after every 24 h. Scale bar: 400 μm. *P < 0.05, **P < 0.001, ***P < 0.0001, 2‐tailed Student's *t* test (Red * – HCT116 C7 and Black * – HCT116 C9).

We also constructed a GFP‐tagged version of the MLL1‐ZC3H13 chimera to monitor its expression and localization pattern. Much like the localization pattern of wild‐type MLL1 and other known MLL1‐fusion proteins (Caslini *et al*., 2000; Manara *et al*., 2014), the MLL1‐ZC3H13‐GFP fusion resided mainly inside the nuclei of HCT116 cells, with some nuclear granule‐like structures, as previously observed for similar MLL1 fusions (Caslini *et al*., 2000; Manara *et al*., 2014; Marschalek, 2016) (Fig. 4A). An increase in nuclear size was consistently observed in cells expressing GFP‐tagged MLL1‐ZC3H13 fusion protein. In addition, over 50% of the cells expressing MLL1‐ZC3H13‐GFP exhibited morphological abnormalities including multi‐nucleation (Fig. 4B,C) and lagging chromosomes (Fig. S6B). As the changes in nuclear areas are more likely to reflect large genomic alterations, such as those associated with tetraploidy (or beyond), this phenotype implied that the expression of the MLL1‐ZC3H13 fusion protein induces CIN. To address this possibility, we used flow cytometry to monitor cell cycle progression of the two clones (clones 7 and 9) expressing the fusion chimera and found a twofold increase in apoptotic population and aneuploidy (Fig. 4D,E). A significant time‐dependent increase in apoptotic cell population was also observed (Fig. S7). Interestingly, the number of cells with aneuploidy in the MLL1‐ZC3H13 cells also gradually decreased with time, which could be attributed to the increase in apoptosis, as it is well known that aneuploidy often leads to cell death (Kops *et al*., 2005; London and Biggins, 2014; Malmanche *et al*., 2006). Consistent with this, we observed an overall reduction in the growth rates of the C7 and C9 clones compared with vector controls (Fig. S8A,B).

To assess whether the expression of MLL1‐ZC3H13 fusion chimera affects mitotic progression, cells were released from a double thymidine block and samples were collected at different time points as represented in the schematic (Fig. 4F). The harvested cells were stained for phospho‐histone H3 positivity to allow examination of mitotic cells by flow cytometry. Mitotic entry in vector control cells was observed around 9–12 h after release; however, in the two selected clones, we observed a significant decrease in mitotic cell population compared with the vector controls (Figs 4F and S8C). Given that we observed mitotic defects, including aneuploidy in MLL‐ZC3H13 fusion, the reduced mitotic population in C7 and C9 clones may indicate an ineffective mitotic checkpoint in these clones. As defects in the mitotic checkpoints also generate aneuploidy and might facilitate CIN (Kops *et al*., 2005; London and Biggins, 2014; Malmanche *et al*., 2006), our results indicate that expression of MLL1‐ZC3H13 induces CIN.

### 3.5 MLL1‐ZC3H13 fusion enhances pro‐tumorigenic properties of HCT116 colorectal cells

**Figure 5:**
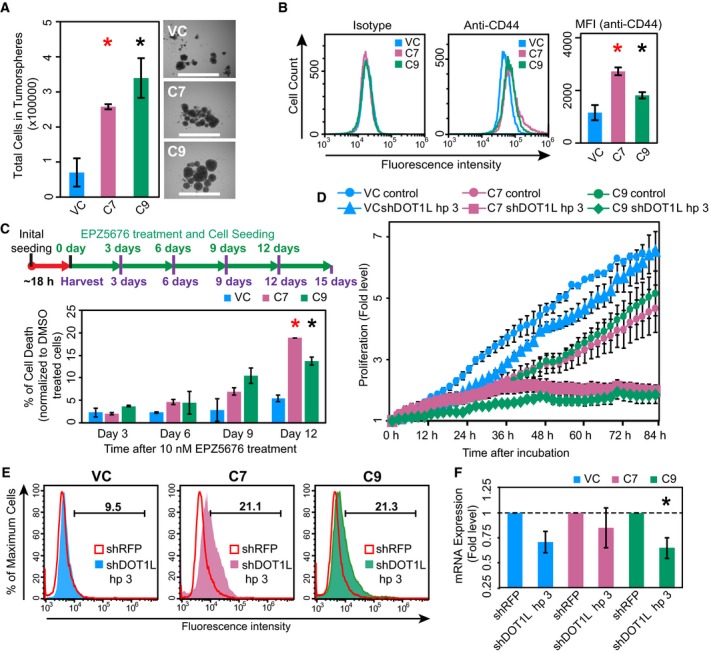
Assays to demonstrate tumorigenesis potential and collateral lethality. (A) Tumorsphere‐forming capacity of the C7, C9, and vector control was assessed by growing the cells in low adhesion plates along with tumorsphere medium for 7 days, after which the cells were collected and quantified. Representative images of tumorspheres after 7 days of incubation. Scale bar: 1000 μm. (B) CD44 expression in C7, C9, and vector control cells was assessed by flow cytometry and their histograms were overlaid for test and isotype control. Median fluorescent intensity values calculated are plotted as bar chart. (C) Schematic representation of the drug Pinometostat (EPZ‐5676) treatment. Graphical representation of percentage of cell death following normalization with DMSO‐treated cells in C7, C9, and vector control as assessed by resazurin viability assay following drug treatment using 10 nm EPZ‐5676 at various time points. *P < 0.05, **P < 0.001, ***P < 0.0001, 2‐tailed Student's *t* test (Red * – HCT116 C7 and Black * – HCT116 C9). (D) Graphical representation of the fold level changes in proliferation between C7, C9, and vector control as assessed by automated live‐cell analysis system (incucyte®s3), (E) Histogram showing live‐dead analysis using 7AAD staining following shRNA silencing of C7, C9, and vector control using shDOT1L hairpin 3 (HCT116 C7 – Pink; HCT116 C9 – Green; Vector control – Blue) and overlaid with RFP hairpin mix (Red inlay). Representative images show mean percentage values of three experiments, (F) Bar graph showing decrease in mRNA expression of *DOT1L* in C7, C9, and vector control following shRNA silencing using RFP (negative control) and DOT1L hairpins (Hp 3) and determined by qRT‐PCR. *P < 0.05, 2‐tailed Student's *t* test (Black * – HCT116 C9). The error bars in all the subfigures represent the standard error of the mean (SE).

Our experiments indicated that the MLL1‐ZC3H13 chimera induced CIN and aneuploidy. We therefore attempted to assess the tumorigenic potential of the MLL1‐ZC3H13 fusion. Anchorage‐independent growth is the ability of transformed cells to grow independently of a solid substrate, and is a well‐established characteristic of cells that undergo malignant transformation (Borowicz *et al*., 2014). Interestingly, our studies demonstrated that cells expressing the fusion chimera formed an increased number of large‐sized colonies compared with control cells, when cultured in substrate‐free conditions, on soft agar (Fig. 4G). In contrast, the wound‐healing assay (Liang *et al*., 2007) did not show any marked difference in the migration activities of these cells (Fig. 4H). We also monitored the effect of the MLL1‐ZC3H13 fusion on tumorsphere formation. Remarkably, both C7 and C9 cells formed larger tumorspheres than the vector control (Fig. 5A). As our analysis of tumorspheres indicated that the fusion protein might actually help the expansion of cancer stem cells (CSCs), we further assessed this by flow cytometry using anti‐CD44 antibody. Increased CD44 expression has been shown to enhance CSC properties in colon cancer cells (Cho *et al*., 2012; Keysar and Jimeno, 2010). Moreover, the importance of CD44 in colon cancer has been shown to affect tumorigenicity and stemness, and be of clinical and prognostic importance (Du *et al*., 2008; Xia and Xu, 2016; Yan *et al*., 2015; Zhou *et al*., 2016). We found the expression of MLL1‐ZC3H13 fusion increased the level of CD44, very similarly to studies showing truncated MLL fusion proteins, which can immortalize and enhance the self‐renewal potential of CSCs (Fig. 5B) (Martin *et al*., 2003; Ono *et al*., 2013).

MLL1 fusions have been shown to recruit DOT1L, a histone 3 lysine 79 (H3K79) methyltransferase and epigenetic marker of leukemic stem cells which has been implicated in the development of leukemia (Bernt *et al*., 2011). Because we observed MLL‐ZC3H13 fusion affects stemness marker CD44 and potentiates the proliferation of tumorspheres, we hypothesized that MLL1‐ZC3H13 fusion may require DOT1L function. DOT1L requires specific MLL1 fusions that provide either a docking site or facilitate its recruitment (Shen *et al*., 2013; Yokoyama, 2017), but DOT1L is also involved in the regulation of telomeric silencing, development, cell cycle checkpoint, and transcription (Park *et al*., 2010; Wong *et al*., 2015). Therefore, we performed a drug inhibition assay using Pinometostat (EPZ5676), a known DOT1L inhibitor (Daigle *et al*., 2013), and found that the C7 and C9 clones are more sensitive than the vector control (Fig. 5C and Fig. S9A,B). Although, C7 and C9 clones have growth defects by themselves, we found an additional 15% increase in cell death upon EPZ5676 treatment (Fig. 5C). This is intriguing because previous work has shown that it is only certain fusions of MLL1, like the ones with AF4 and ENL, which are able to recruit DOT1L and we do not know whether our fusion construct functions in the same way. Therefore, we used shRNA targeting *DOT1L* and assessed viability in C7, C9, and vector control cells by proliferation studies in automated live‐cell analyzer (incucyte®s3) and by live‐dead staining with 7AAD (Figs  5D,E and S9C). These experiments demonstrated that knockdown of DOT1L specifically affected C7 and C9 clones and led to further decrease in their viability. We tested three independent shRNA and chose the one that had the highest efficiency in silencing of DOT1L (Fig. S9C). The effect of shRNA to knockdown DOT1L was confirmed by reduced mRNA expression using qRT‐PCR (Figs 5F and S9D). Taken together our findings demonstrate the tumorigenic property of MLL‐ZC3H13 fusion and a potential collateral lethality (Muller *et al*., 2015) between the DOT1L‐associated functions and MLL1 fusions in HCT116 cells.

## 4 Discussion

MLL1 fusions occur with high frequency, and the role and mechanism of these fusion proteins exerting their oncogenic function in leukemia has been well studied (Marschalek, 2011, 2016; Rao and Dou, 2015; Slany, 2016). Here we demonstrated that MLL1‐fusion can also occur in prostate tumors, albeit with low frequency (1.19% of all cases). That being said, we performed our analyses using the raw TCGA data derived from total tumor cell populations, where natural heterogeneity is likely to mask the presence of MLL1 fusions in smaller cell groups, such as CSC for example. It is worth mentioning here that even the relatively well‐studied EML‐ALK fusion in non‐small cell lung cancer (Morodomi *et al*., 2014) or PML‐RAR fusion in acute myelogenous leukemia (Saeed *et al*., 2011) is only found in 2–5% or 6–10% of these cancers, respectively. Moreover, identifying these fusion events is not as straightforward as identifying a genetic mutation. The raw sequence needs to be scanned across the entire genome for the presence of the fusion, as they may occur at any locus and directionality, which makes this computationally demanding. Therefore, the development of algorithms to detect these fusions efficiently will greatly benefit robust identification of fusion proteins. Thus, tracking of these chromosomal rearrangements requires an in‐depth analysis of the transcriptome data. Single cell sequencing will likely reveal the dynamic range of MLL1 fusions in solid tumors. Interestingly, until now only one report has indicated the existence of MLL1 fusions in any solid tumors, and this has also been found in prostate tumors (Chowdry *et al*., 2016). In support of our findings, recent studies have reported that Menin, a critical co‐activator of androgen receptor (AR), along with MLL complex is recruited to the promoter of AR by BAP18 to promote its transcriptional activation and drive prostate cancers (Iba *et al*., 1985; Malik *et al*., 2015; Shaw *et al*., 2018). Since Menin binds to the N‐terminal of MLL (Thiel *et al*., 2012) and most truncations of MLL1 occur at the C‐terminus (Meyer *et al*., 2018), MLL‐Menin binding is possible even in fusion proteins with truncated MLL (Yokoyama *et al*., 2004). Thus, there is a viable option for fusions or truncated forms of MLL1 to promote prostate cancer progression. Further mechanistic studies are required to investigate this.

Our complete analyses of the raw sequence data from the prostate tumors identified six patients that have lost the functional SET domain of MLL1, resulting in the formation of MLL1‐TTC36 fusions. While this could be due to alternative or trans‐splicing events, as *TTC36* is also located on chromosome 11, it is interesting to note that 12 of the known fusions of MLL1 reside on chromosome 11 (Meyer *et al*., 2018). The presence of the MLL1‐TTC36 fusion in one of the normal samples may indicate a significant risk for as yet undetected prostate cancer in this individual, since truncated MLL fusions are known to have an oncogenic effect (Dobson *et al*., 2000; Wachter *et al*., 2014). It is interesting to note that *TTC36* (also called HBP21) encodes a poorly characterized tumor suppressor whose loss of function has been associated with hepatocellular carcinoma (Jiang *et al*., 2015). Thus, MLL1‐TTC36 and MLL1‐REPS2 fusions warrant further investigations for their role in tumorigenesis.

Chromosomal rearrangements provide a unique opportunity selectively to kill cancer cells, and this MLL1‐fusion is no exception. Cells require the wild‐type *MLL1* gene for viability in the presence of a heterozygous MLL1‐fusion gene, as antisense‐dependent knockdown of wild‐type MLL1 reduces tumor growth and angiogenesis *in vivo* (Ansari *et al*., 2013). Thus, targeting MLL1 *per se* is not a viable option, as it will affect the normal cells as well. An alternative mechanism is to disrupt the transcriptional program of the MLL1 fusions. Here we demonstrate that inhibition of the H3K79 methyltransferase DOT1L selectively kills tumor cells that exhibit this chromosomal rearrangement. Overall, our study demonstrates that MLL1 fusions may also be prevalent in solid tumors, and the DOT1L‐dependent treatment strategy used in leukemia should be applicable to these tumors.

Although we focused on MLL1‐ZC3H13 fusion as a model, we did not identify MLL1‐ZC3H13 fusion in the TCGA prostate cancer dataset. Nevertheless, given the nature of data (whole population vs single cell) used to analyze the depth of heterogeneity, one cannot exclude the presence of this fusion. We focused on *ZC3H13* because Vogelstein and colleagues proposed that *ZC3H13* might be an ortholog of the yeast securin (*ySecurin*) gene *PDS1* (Wang *et al*., 2004). Since there is no sequence similarity between the human securin gene *PTTG1* (*hSecurin*) and *ySecurin,* they were considered functional orthologs. This was in agreement with the synthetic lethal relation reported between *ZC3H13* and *hSecurin*, as they could have functionally diverged over evolution with some overlapping functions (Vizeacoumar *et al*., 2013). Consistent with this, we observed a negative correlation in expression between *hSecurin* and *ZC3H13* across multiple cancers including samples from breast, kidney, and ovarian cancer patients (Fig. S10A). However, introduction of two different alternative splice variants of *ZC3H13* to complement the growth defect of a yeast mutant defective in *PDS1*, did not rescue its growth (Fig. S10B), indicating either that *ZC3H13* is not a *PDS1* ortholog or that *ZC3H13* and *PDS1* have diverged in function in this cross‐species assay.

## 5 Conclusion

We have engineered a model system that could be used to study MLL1 fusions in solid tumors. In particular, our system captures most of the known properties exhibited by these fusion chimeras and therefore can be exploited further to understand the mechanism of MLL1 fusions in solid tumors. While more work needs to be done to investigate the prevalence of fusion proteins in solid tumors, our work represents a first step in this direction. We also anticipate that future work in single‐cell genomics will unravel these rare incidences of chromosomal rearrangements.

## Conflict of interest

The authors declare no conflict of interest.

## Author contributions

Conceptualization – SP, FSV and FJV; methodology – SP, FSV, KKB, FQ, BMT, PCS and HL; investigation – SP, FSV and FJV; writing original draft – SP and FJV; writing review and editing – SP, FSV, KKB, FQ, BMT, PCS, MFI, CEC, DDM, MCU, PCS, YW, KB, AF and HL; funding acquisition – FJV; resources – MFI, CEC, DDM, MCU, PCS, YW, KB, AF, HL and FJV; supervision – FJV and HL.

## Supporting information


**Fig. S1.** Comparison of MLL1‐fusion partner expression in various cancers.
**Fig. S2.** Significance of MLL1‐fusion partners in solid and liquid tumors.
**Fig. S3.** *In silico* analysis of predicted function of MLL1‐fusion partners represented using Cytoscape.
**Fig. S4.** Genetic variations in MLL1‐fusion partners using cBioPortal.
**Fig. S5.** Copy Number Variations in various cancers.
**Fig. S6.** Clone validation and characteristics.
**Fig. S7.** Cell cycle analysis using propidium iodide.
**Fig. S8.** Cell cycle and division characteristics of clones.
**Fig. S9.** Drug inhibition assay.
**Fig. S10.** Correlation and complementation analyses of ZC3H13.

**Table S1.** Pathway‐specific GO terms used.

## Data Availability

Publicly available datasets used in the study has been cited as required.
