# Supplementary figures and images for: Molecular characterization of an MLL1 fusion and its role in chromosomal instability
Source: Mol Oncol. 2018 Dec 31;13(2):422–40. doi: 10.1002/1878-0261.12423 (PMC6360371; doi:10.1002/1878-0261.12423)

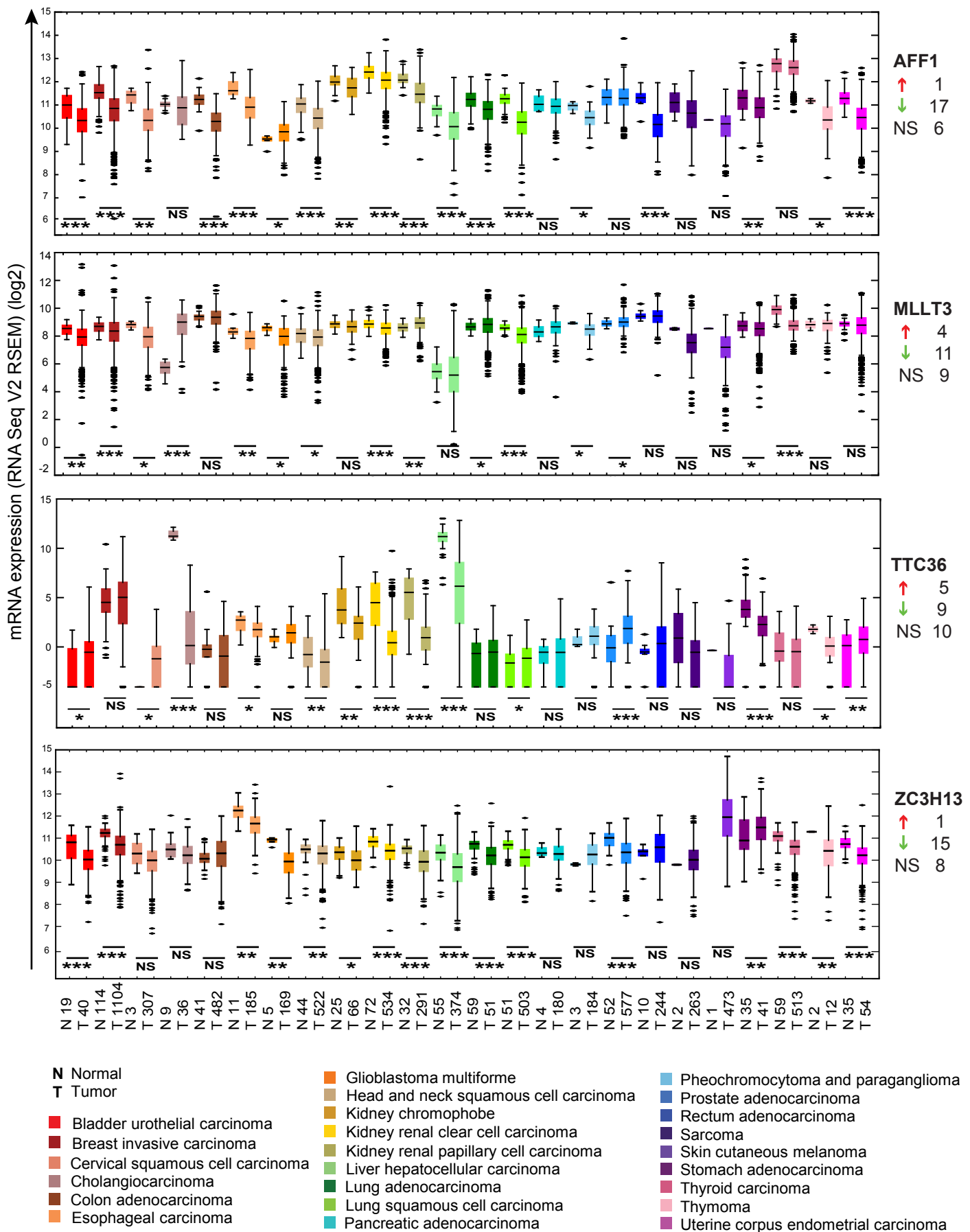

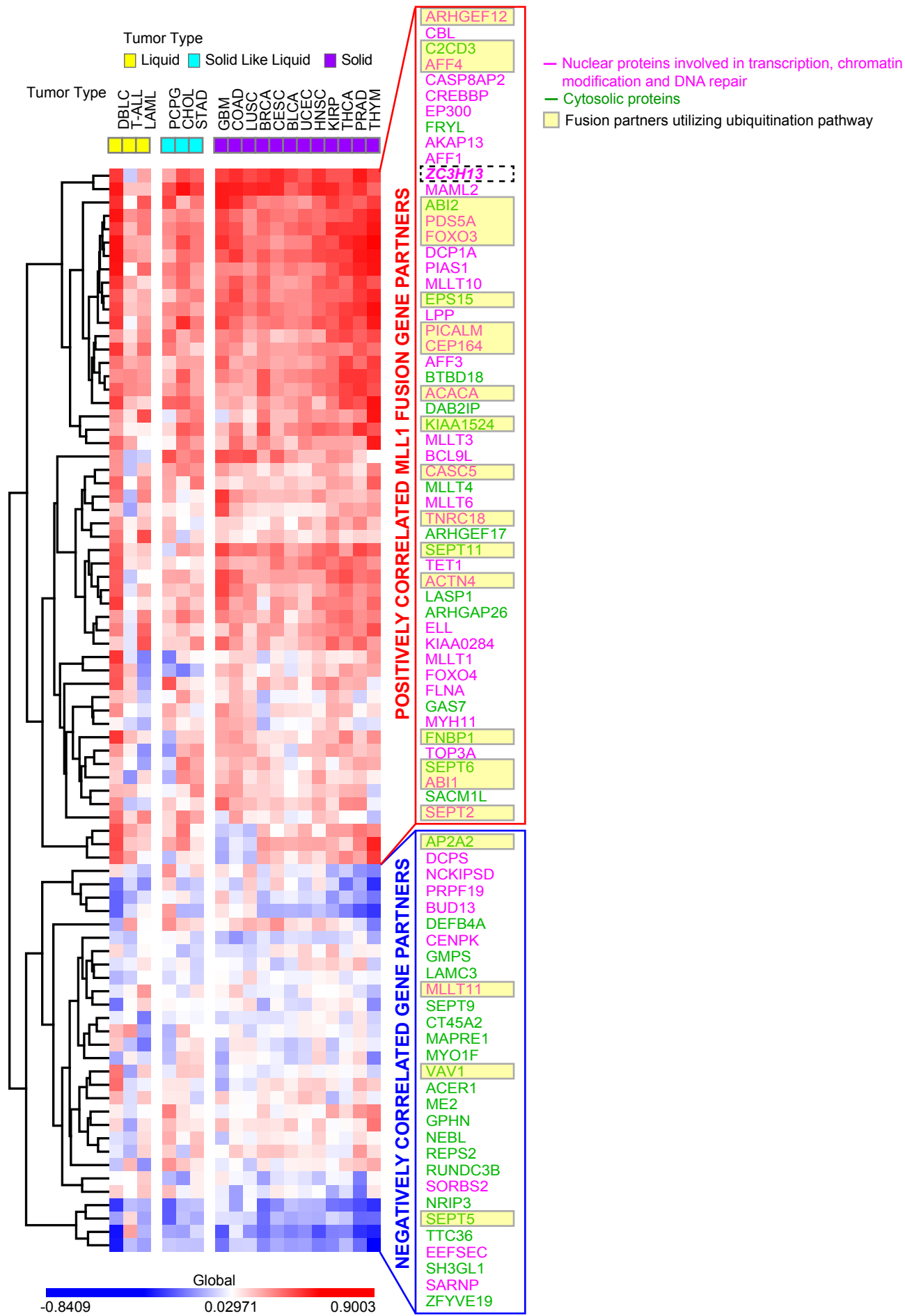

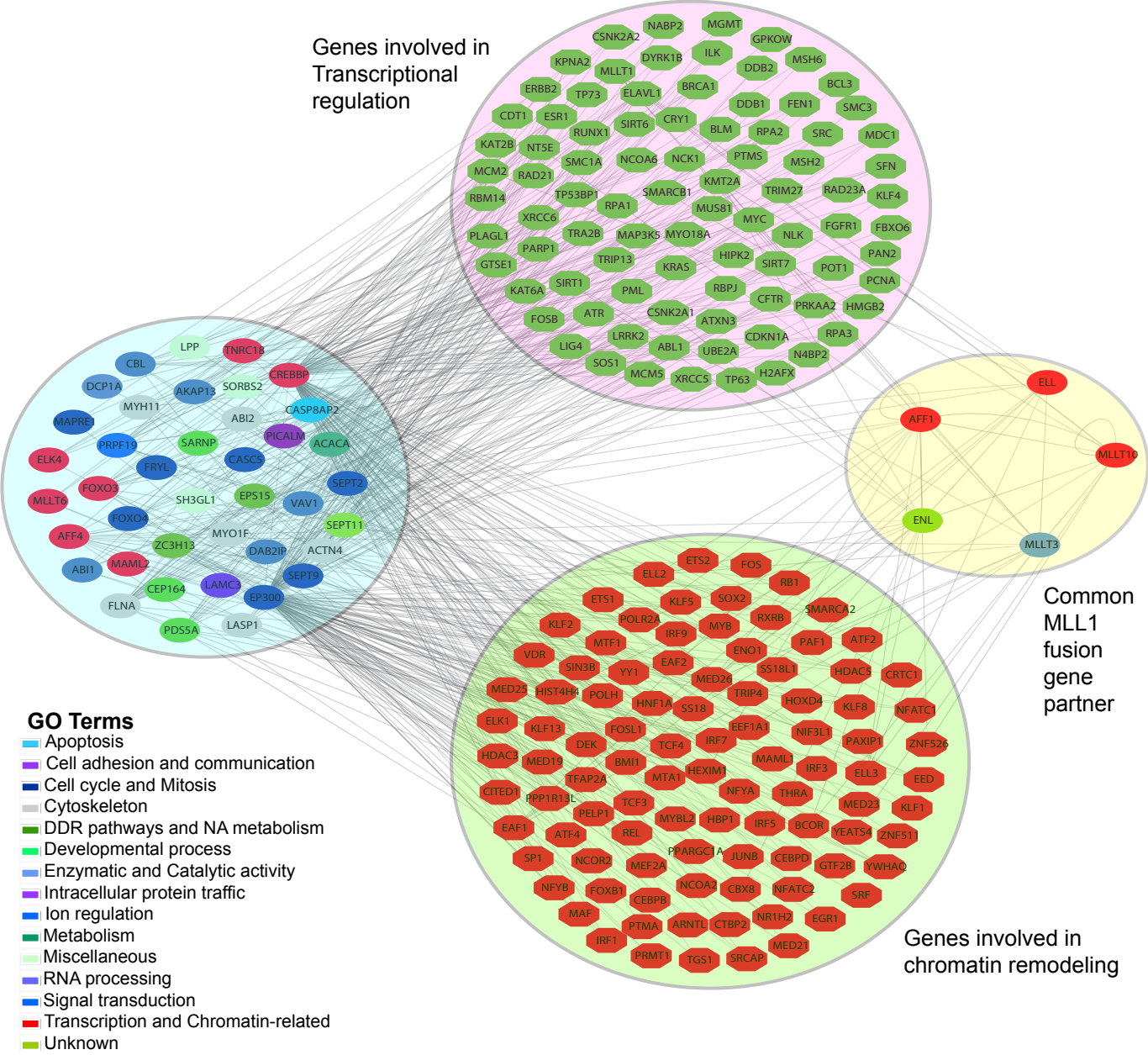

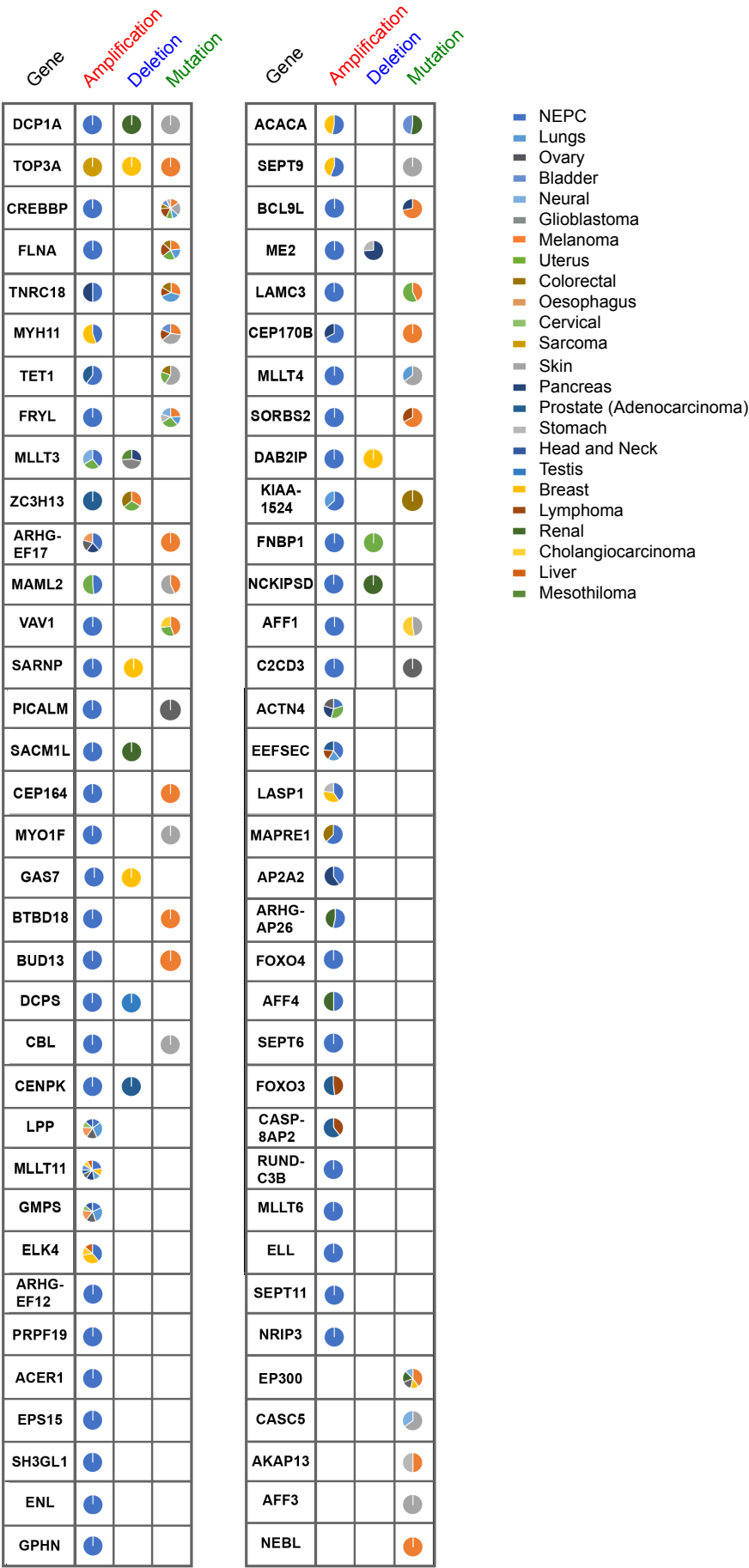

Amplification Gain Shallow Deletion Deep Deletion

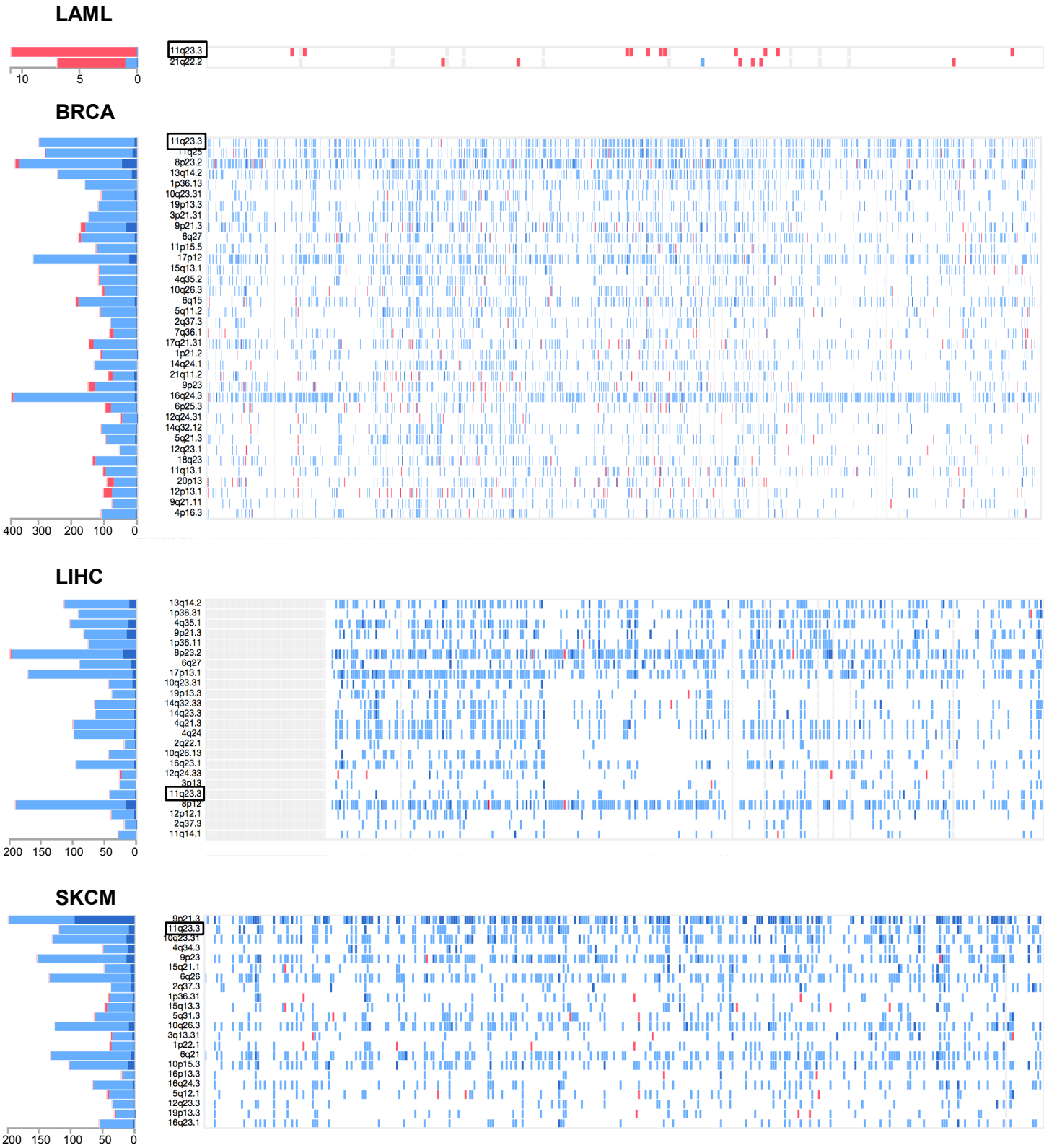

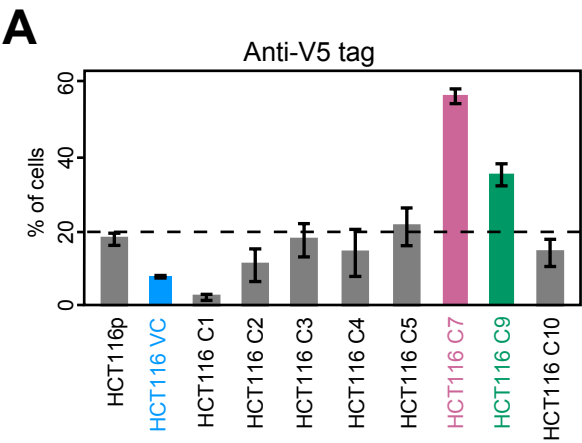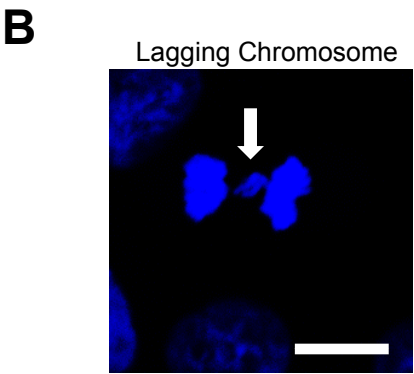

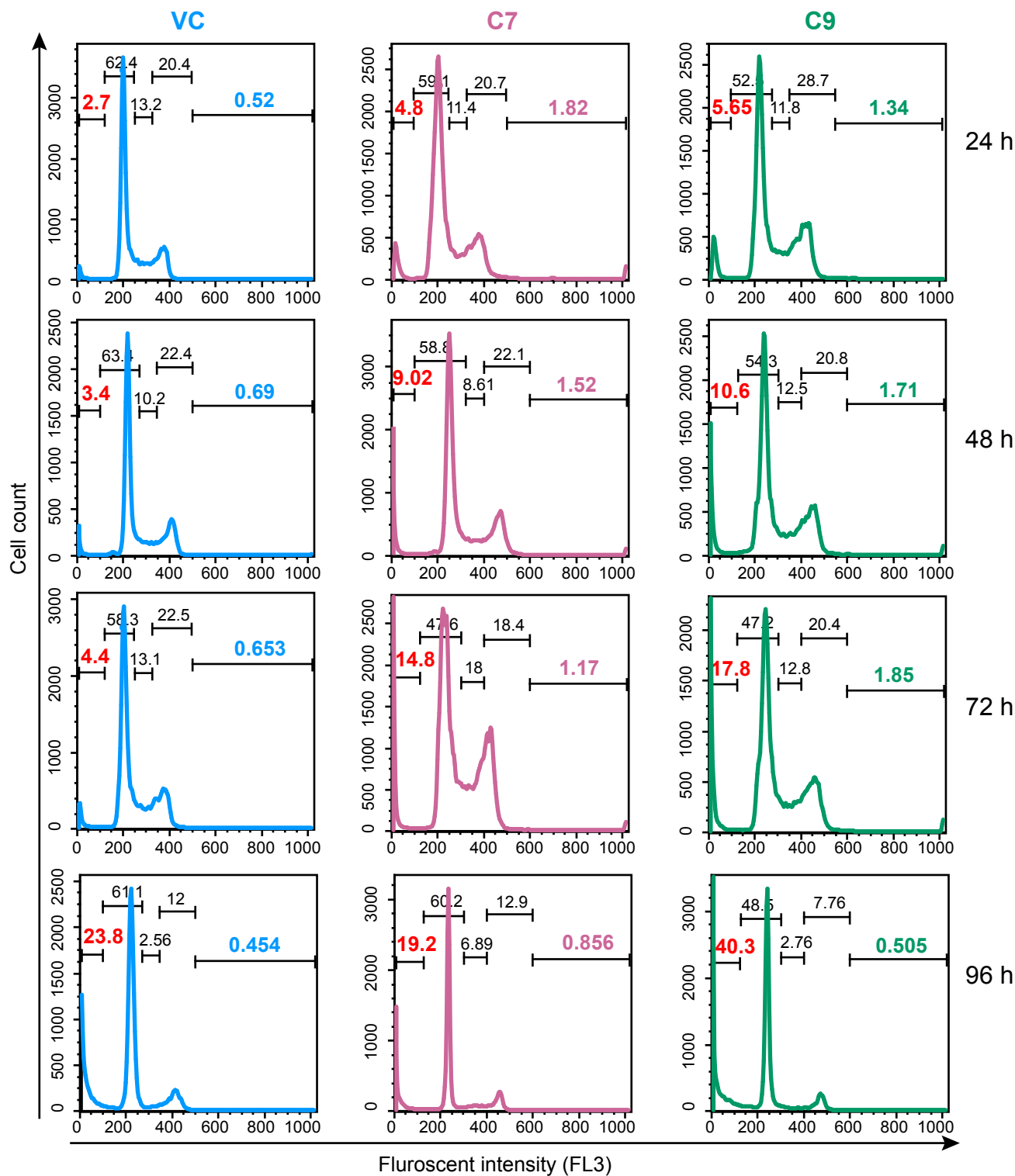

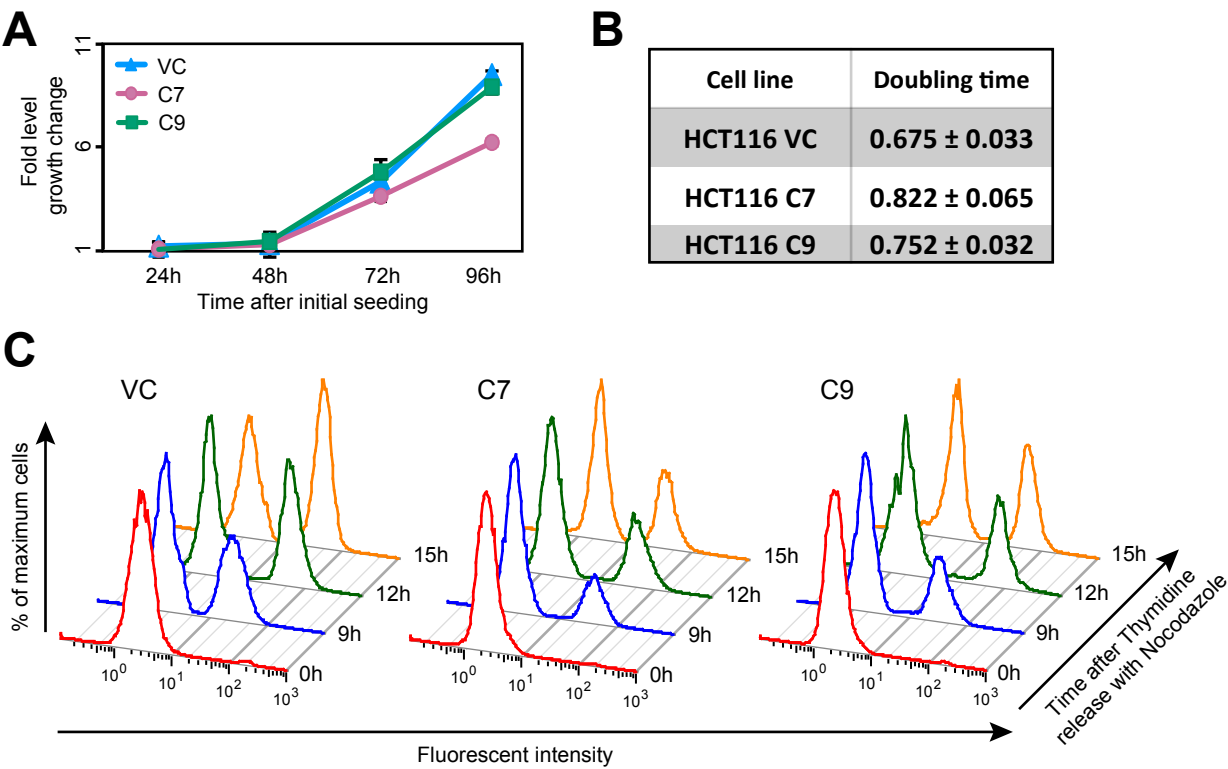

**A**

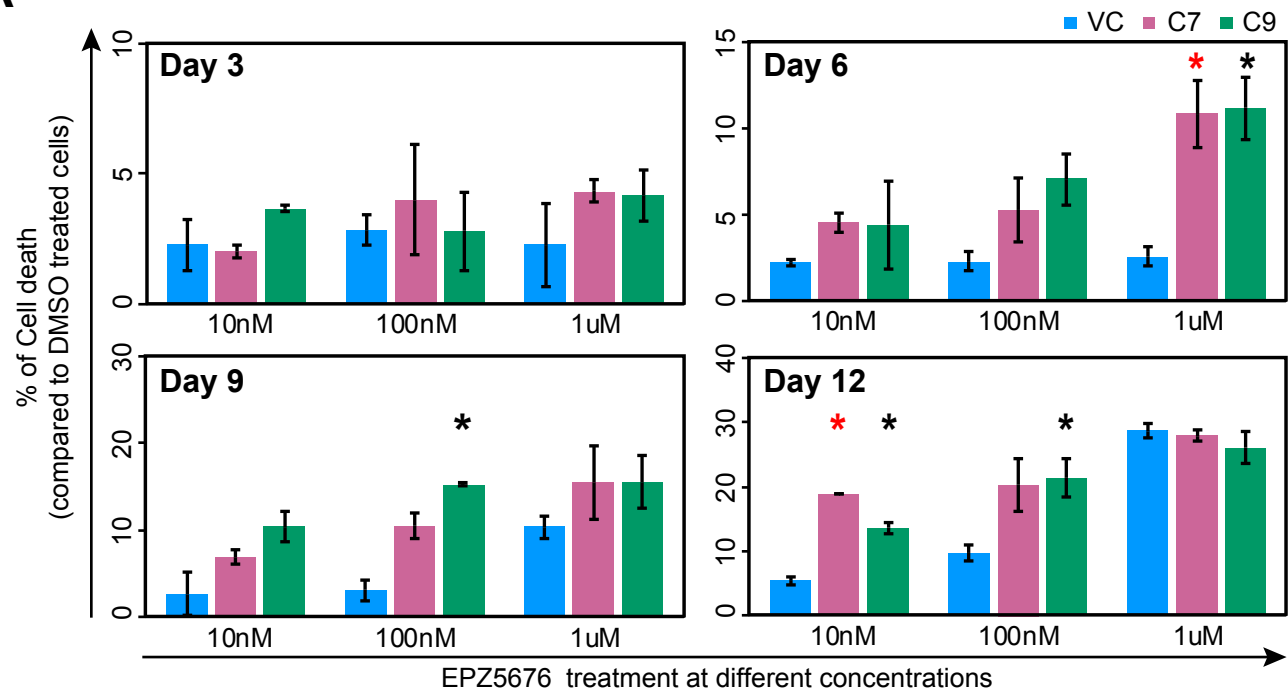

**B**

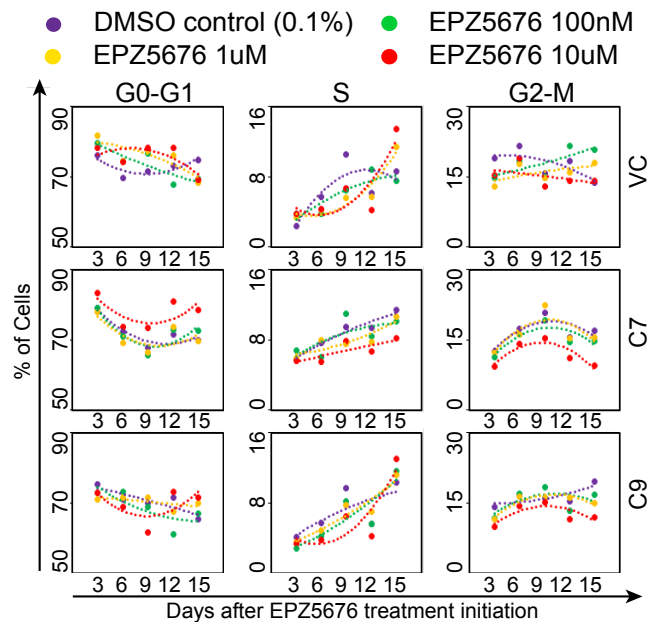

**C**

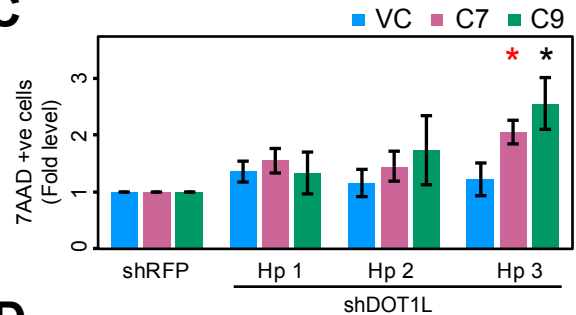

**D**

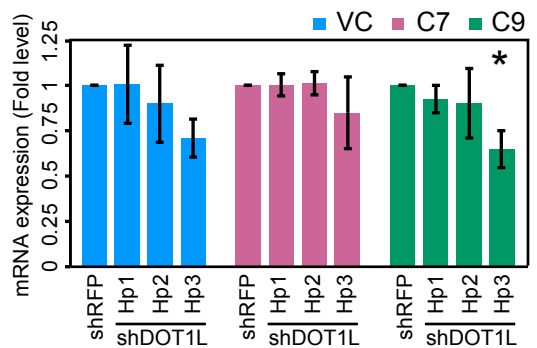

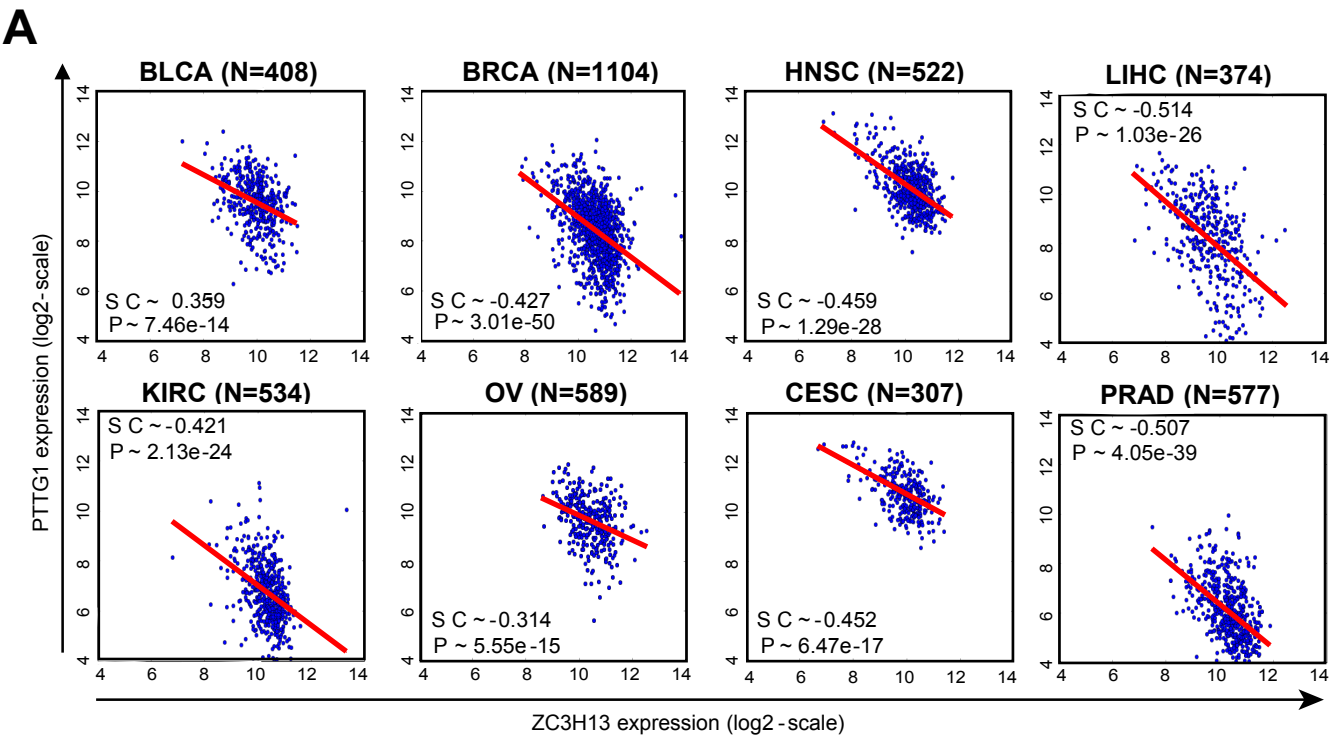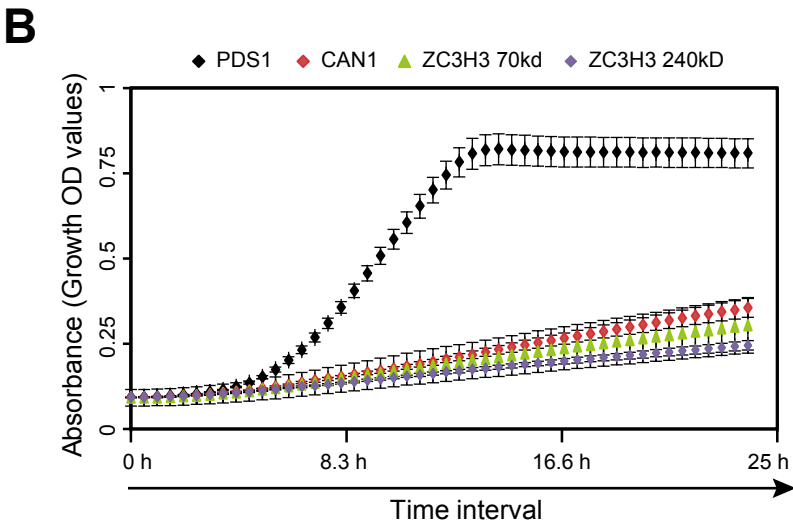

Supplement: Supplementary file 1 — Fig. S1. Comparison of MLL1‐fusion partner expression in various cancers. Fig. S2. Significance of MLL1‐fusion partners in solid and liquid tumors. Fig. S3. In silico analysis of predicted function of MLL1‐fusion partners represented using Cytoscape. Fig. S4. Genetic variations in MLL1‐fusion partners using cBioPortal. Fig. S5. Copy Number Variations in various cancers. Fig. S6. Clone validation and characteristics. Fig. S7. Cell cycle analysis using propidium iodide. Fig. S8. Cell cycle and division characteristics of clones. Fig. S9. Drug inhibition assay. Fig. S10. Correlation and complementation analyses of ZC3H13. [file MOL2-13-422-s001.pdf]
