# Supplementary material for: Molecular characterization of an MLL1 fusion and its role in chromosomal instability
Source: Mol Oncol. 2018 Dec 31;13(2):422–40. doi: 10.1002/1878-0261.12423 (PMC6360371; doi:10.1002/1878-0261.12423)
Supplement: Supplementary file 2 — Table S1. Pathway‐specific GO terms used. [file MOL2-13-422-s002.pdf]

Supplementary Table S1. Pathway specific GO terms used.

TGF-beta Receptor Signaling Pathway			
	Entrez Gene ID	Gene Symbol	Gene Name
1	2099	ESR1	estrogen receptor 1
2	2113	ETS1	ETS proto-oncogene 1, transcription factor
3	4205	MEF2A	myocyte enhancer factor 2A
4	163	AP2B1	adaptor related protein complex 2 beta 1 subunit
5	10413	YAP1	Yes associated protein 1
6	51433	ANAPC5	anaphase promoting complex subunit 5
7	51434	ANAPC7	anaphase promoting complex subunit 7
8	2308	FOXO1	forkhead box O1
9	2309	FOXO3	forkhead box O3
10	8454	CUL1	cullin 1
11	2353	FOS	Fos proto-oncogene, AP-1 transcription factor subunit
12	2354	FOSB	FosB proto-oncogene, AP-1 transcription factor subunit
13	4435	CITED1	Cbp/p300 interacting transactivator with Glu/Asp rich carboxy-terminal domain 1
14	367	AR	androgen receptor
15	409	ARRB2	arrestin beta 2
16	22938	SNW1	SNW domain containing 1
17	8648	NCOA1	nuclear receptor coactivator 1
18	4609	MYC	v-myc avian myelocytomatosis viral oncogene homolog
19	6667	SP1	Sp1 transcription factor
20	55914	ERBIN	erbb2 interacting protein
21	6794	STK11	serine/threonine kinase 11
22	8850	KAT2B	lysine acetyltransferase 2B
23	8881	CDC16	cell division cycle 16
24	4800	NFYA	nuclear transcription factor Y subunit alpha
25	4801	NFYB	nuclear transcription factor Y subunit beta
26	10987	COPS5	COP9 signalosome subunit 5
27	8945	BTRC	beta-transducin repeat containing E3 ubiquitin protein ligase
28	6935	ZEB1	zinc finger E-box binding homeobox 1
29	860	RUNX2	runt related transcription factor 2
30	9146	HGS	hepatocyte growth factor-regulated tyrosine kinase substrate
31	960	CD44	CD44 molecule (Indian blood group)
32	996	CDC27	cell division cycle 27
33	7157	TP53	tumor protein p53
34	1017	CDK2	cyclin dependent kinase 2

35	3065	HDAC1	histone deacetylase 1
36	7161	TP73	tumor protein p73
37	1021	CDK6	cyclin dependent kinase 6
38	1026	CDKN1A	cyclin dependent kinase inhibitor 1A
39	7321	UBE2D1	ubiquitin conjugating enzyme E2 D1
40	7322	UBE2D2	ubiquitin conjugating enzyme E2 D2
41	7323	UBE2D3	ubiquitin conjugating enzyme E2 D3
42	7341	SUMO1	small ubiquitin-like modifier 1
43	5295	PIK3R1	phosphoinositide-3-kinase regulatory subunit 1
44	5296	PIK3R2	phosphoinositide-3-kinase regulatory subunit 2
45	29882	ANAPC2	anaphase promoting complex subunit 2
46	3312	HSPA8	heat shock protein family A (Hsp70) member 8
47	7421	VDR	vitamin D (1,25- dihydroxyvitamin D3) receptor
48	7514	XPO1	exportin 1
49	1386	ATF2	activating transcription factor 2
50	1387	CREBBP	CREB binding protein
51	1432	MAPK14	mitogen-activated protein kinase 14
52	5576	PRKAR2A	protein kinase cAMP-dependent type II regulatory subunit alpha
53	5580	PRKCD	protein kinase C delta
54	1499	CTNNB1	catenin beta 1
55	5599	MAPK8	mitogen-activated protein kinase 8
56	1601	DAB2	DAB2, clathrin adaptor protein
57	1616	DAXX	death domain associated protein
58	3725	JUN	Jun proto-oncogene, AP-1 transcription factor subunit
59	3726	JUNB	JunB proto-oncogene, AP-1 transcription factor subunit
60	5925	RB1	RB transcriptional corepressor 1
61	57154	SMURF1	SMAD specific E3 ubiquitin protein ligase 1
62	79753	SNIP1	Smad nuclear interacting protein 1
63	2033	EP300	E1A binding protein p300
64	4087	SMAD2	SMAD family member 2
65	4088	SMAD3	SMAD family member 3
66	4089	SMAD4	SMAD family member 4
67	4092	SMAD7	SMAD family member 7

B Cell Receptor Signaling Pathway			
	**Entrez Gene ID**	**Gene Symbol**	**Gene Name**
1	6195	RPS6KA1	ribosomal protein S6 kinase A1
2	2185	PTK2B	protein tyrosine kinase 2 beta
3	207	AKT1	AKT serine/threonine kinase 1
4	2308	FOXO1	forkhead box O1
5	6452	SH3BP2	SH3 domain binding protein 2
6	6464	SHC1	SHC adaptor protein 1
7	8517	IKBKG	inhibitor of nuclear factor kappa B kinase subunit gamma
8	387	RHOA	ras homolog family member A
9	2534	FYN	FYN proto-oncogene, Src family tyrosine kinase
10	6654	SOS1	SOS Ras/Rac guanine nucleotide exchange factor 1
11	4690	NCK1	NCK adaptor protein 1
12	604	BCL6	B-cell CLL/lymphoma 6
13	6772	STAT1	signal transducer and activator of transcription 1
14	6774	STAT3	signal transducer and activator of transcription 3
15	4772	NFATC1	nuclear factor of activated T-cells 1
16	4773	NFATC2	nuclear factor of activated T-cells 2
17	695	BTK	Bruton tyrosine kinase
18	6850	SYK	spleen associated tyrosine kinase
19	2885	GRB2	growth factor receptor bound protein 2
20	7006	TEC	tec protein tyrosine kinase
21	867	CBL	Cbl proto-oncogene
22	868	CBLB	Cbl proto-oncogene B
23	890	CCNA2	cyclin A2
24	921	CD5	CD5 molecule
25	975	CD81	CD81 molecule
26	3055	HCK	HCK proto-oncogene, Src family tyrosine kinase
27	1017	CDK2	cyclin dependent kinase 2
28	1021	CDK6	cyclin dependent kinase 6
29	29760	BLNK	B-cell linker
30	3190	HNRNPK	heterogeneous nuclear ribonucleoprotein K
31	1147	CHUK	conserved helix-loop-helix ubiquitous kinase
32	5295	PIK3R1	phosphoinositide-3-kinase regulatory subunit 1
33	5296	PIK3R2	phosphoinositide-3-kinase regulatory subunit 2
34	5335	PLCG1	phospholipase C gamma 1
35	7454	WAS	Wiskott-Aldrich syndrome
36	1385	CREB1	cAMP responsive element binding protein 1
37	1386	ATF2	activating transcription factor 2

38	7535	ZAP70	zeta chain of T cell receptor associated protein kinase 70
39	1398	CRK	CRK proto-oncogene, adaptor protein
40	1399	CRKL	CRK like proto-oncogene, adaptor protein
41	1432	MAPK14	mitogen-activated protein kinase 14
42	5580	PRKCD	protein kinase C delta
43	5588	PRKCQ	protein kinase C theta
44	5594	MAPK1	mitogen-activated protein kinase 1
45	1499	CTNNB1	catenin beta 1
46	5595	MAPK3	mitogen-activated protein kinase 3
47	3551	IKBKB	inhibitor of nuclear factor kappa B kinase subunit beta
48	5599	MAPK8	mitogen-activated protein kinase 8
49	5604	MAP2K1	mitogen-activated protein kinase kinase 1
50	5747	PTK2	protein tyrosine kinase 2
51	3725	JUN	Jun proto-oncogene, AP-1 transcription factor subunit
52	5777	PTPN6	protein tyrosine phosphatase, non-receptor type 6
53	10014	HDAC5	histone deacetylase 5
54	5925	RB1	RB transcriptional corepressor 1
55	5966	REL	REL proto-oncogene, NF-kB subunit
56	5970	RELA	RELA proto-oncogene, NF-kB subunit
57	3937	LCP2	lymphocyte cytosolic protein 2
58	10092	ARPC5	actin related protein 2/3 complex subunit 5
59	10093	ARPC4	actin related protein 2/3 complex subunit 4
60	10094	ARPC3	actin related protein 2/3 complex subunit 3
61	10096	ACTR3	ARP3 actin related protein 3 homolog
62	10097	ACTR2	ARP2 actin related protein 2 homolog
63	2002	ELK1	ELK1, ETS transcription factor
64	4067	LYN	LYN proto-oncogene, Src family tyrosine kinase

EGFR1 Signaling Pathway			
	**Entrez Gene ID**	**Gene Symbol**	**Gene Name**
1	2059	EPS8	epidermal growth factor receptor pathway substrate 8
2	2060	EPS15	epidermal growth factor receptor pathway substrate 15
3	10253	SPRY2	sprouty RTK signaling antagonist 2
4	6195	RPS6KA1	ribosomal protein S6 kinase A1
5	8290	HIST3H3	histone cluster 3 H3
6	2185	PTK2B	protein tyrosine kinase 2 beta
7	160	AP2A1	adaptor related protein complex 2 alpha 1 subunit
8	207	AKT1	AKT serine/threonine kinase 1
9	8440	NCK2	NCK adaptor protein 2
10	2308	FOXO1	forkhead box O1
11	2353	FOS	Fos proto-oncogene, AP-1 transcription factor subunit
12	6456	SH3GL2	SH3 domain containing GRB2 like 2, endophilin A1
13	6457	SH3GL3	SH3 domain containing GRB2 like 3, endophilin A3
14	6464	SHC1	SHC adaptor protein 1
15	6633	SNRPD2	small nuclear ribonucleoprotein D2 polypeptide
16	6654	SOS1	SOS Ras/Rac guanine nucleotide exchange factor 1
17	4609	MYC	v-myc avian myelocytomatosis viral oncogene homolog
18	6667	SP1	Sp1 transcription factor
19	6714	SRC	SRC proto-oncogene, non-receptor tyrosine kinase
20	4690	NCK1	NCK adaptor protein 1
21	6772	STAT1	signal transducer and activator of transcription 1
22	6773	STAT2	signal transducer and activator of transcription 2
23	6774	STAT3	signal transducer and activator of transcription 3
24	6776	STAT5A	signal transducer and activator of transcription 5A
25	8976	WASL	Wiskott-Aldrich syndrome like
26	2885	GRB2	growth factor receptor bound protein 2
27	867	CBL	Cbl proto-oncogene
28	868	CBLB	Cbl proto-oncogene B
29	998	CDC42	cell division cycle 42
30	3064	HTT	huntingtin
31	3065	HDAC1	histone deacetylase 1
32	1051	CEBPB	CCAAT/enhancer binding protein beta
33	9252	RPS6KA5	ribosomal protein S6 kinase A5
34	5295	PIK3R1	phosphoinositide-3-kinase regulatory subunit 1
35	5296	PIK3R2	phosphoinositide-3-kinase regulatory subunit 2
36	3265	HRAS	HRas proto-oncogene, GTPase
37	5335	PLCG1	phospholipase C gamma 1

38	5339	PLEC	plectin
39	29924	EPN1	epsin 1
40	5359	PLSCR1	phospholipid scramblase 1
41	30011	SH3KBP1	SH3 domain containing kinase binding protein 1
42	1385	CREB1	cAMP responsive element binding protein 1
43	7529	YWHAB	tyrosine 3-monooxygenase/tryptophan 5-monooxygenase activation protein beta
44	1398	CRK	CRK proto-oncogene, adaptor protein
45	1399	CRKL	CRK like proto-oncogene, adaptor protein
46	1432	MAPK14	mitogen-activated protein kinase 14
47	5578	PRKCA	protein kinase C alpha
48	5594	MAPK1	mitogen-activated protein kinase 1
49	5595	MAPK3	mitogen-activated protein kinase 3
50	5599	MAPK8	mitogen-activated protein kinase 8
51	5604	MAP2K1	mitogen-activated protein kinase kinase 1
52	3636	INPPL1	inositol polyphosphate phosphatase like 1
53	3725	JUN	Jun proto-oncogene, AP-1 transcription factor subunit
54	5777	PTPN6	protein tyrosine phosphatase, non-receptor type 6
55	5829	PXN	Paxillin
56	1759	DNM1	dynamin 1
57	3845	KRAS	KRAS proto-oncogene, GTPase
58	10006	ABI1	abl interactor 1
59	3872	KRT17	keratin 17
60	3875	KRT18	keratin 18
61	1915	EEF1A1	eukaryotic translation elongation factor 1 alpha 1
62	1956	EGFR	epidermal growth factor receptor
63	2002	ELK1	ELK1, ETS transcription factor
64	4087	SMAD2	SMAD family member 2
65	4088	SMAD3	SMAD family member 3

Androgen receptor signaling pathway			
	**Entrez Gene ID**	**Gene Symbol**	**Gene Name**
1	8202	NCOA3	nuclear receptor coactivator 3
2	10273	STUB1	STIP1 homology and U-box containing protein 1
3	4193	MDM2	MDM2 proto-oncogene
4	10401	PIAS3	protein inhibitor of activated STAT 3
5	207	AKT1	AKT serine/threonine kinase 1
6	2274	FHL2	four and a half LIM domains 2
7	10498	CARM1	coactivator associated arginine methyltransferase 1
8	10499	NCOA2	nuclear receptor coactivator 2
9	2308	FOXO1	forkhead box O1
10	2316	FLNA	filamin A
11	10524	KAT5	lysine acetyltransferase 5
12	367	AR	androgen receptor
13	387	RHOA	ras homolog family member A
14	8648	NCOA1	nuclear receptor coactivator 1
15	23028	KDM1A	lysine demethylase 1A
16	6667	SP1	Sp1 transcription factor
17	6714	SRC	SRC proto-oncogene, non-receptor tyrosine kinase
18	573	BAG1	BCL2 associated athanogene 1
19	6774	STAT3	signal transducer and activator of transcription 3
20	8850	KAT2B	lysine acetyltransferase 2B
21	672	BRCA1	BRCA1, DNA repair associated
22	11034	DSTN	destrin, actin depolymerizing factor
23	811	CALR	calreticulin
24	860	RUNX2	runt related transcription factor 2
25	23411	SIRT1	sirtuin 1
26	998	CDC42	cell division cycle 42
27	3065	HDAC1	histone deacetylase 1
28	1026	CDKN1A	cyclin dependent kinase inhibitor 1A
29	7329	UBE2I	ubiquitin conjugating enzyme E2 I
30	7341	SUMO1	small ubiquitin-like modifier 1
31	5295	PIK3R1	phosphoinositide-3-kinase regulatory subunit 1
32	5296	PIK3R2	phosphoinositide-3-kinase regulatory subunit 2
33	1385	CREB1	cAMP responsive element binding protein 1
34	1387	CREBBP	CREB binding protein
35	9611	NCOR1	nuclear receptor corepressor 1
36	9612	NCOR2	nuclear receptor corepressor 2
37	1499	CTNNB1	catenin beta 1

38	1616	DAXX	death domain associated protein
39	5728	PTEN	phosphatase and tensin homolog
40	5747	PTK2	protein tyrosine kinase 2
41	3725	JUN	Jun proto-oncogene, AP-1 transcription factor subunit
42	5925	RB1	RB transcriptional corepressor 1
43	5970	RELA	RELA proto-oncogene, NF-kB subunit
44	1956	EGFR	epidermal growth factor receptor
45	2033	EP300	E1A binding protein p300
46	4088	SMAD3	SMAD family member 3
47	4089	SMAD4	SMAD family member 4
