# Supplementary material for: Molecular characterization of an MLL1 fusion and its role in chromosomal instability
Source: Mol Oncol. 2018 Dec 31;13(2):422–40. doi: 10.1002/1878-0261.12423 (PMC6360371; doi:10.1002/1878-0261.12423)
Supplement: Supplementary file 3 [file MOL2-13-422-s003.docx]

**SUPPLEMENTARY MATERIALS**

**Figure S1. Comparison of MLL1-fusion partner expression in various cancers.** Relative expression of MLL1-fusion partner genes AFF1, MLLT3, TTC36 and ZC3H13 RSEM normalized mRNA in normal and tumor samples of various cancers from the TCGA database (Abbreviation of each cancer is represented as described in the TCGA portal). Red upward arrow and Green downward arrow represents higher and lower gene expression in tumor compared to normal samples respectively. N.S. denotes not significant.

**Figure S2. Significance of MLL1-fusion partners in solid and liquid tumors.** Correlation clustergram of the MLL1-fusion partners derived from analysis of 18 cancer types (Abbreviation of each cancer is represented as described in the TCGA portal). Two distinct classes of fusion partner genes whose expression positively and negatively correlates emerge from the heat map. The fusion partners are highlighted into nuclear (Pink) and cytosolic (Green) proteins and also as proteins involved or requiring ubiquitination pathway (Yellow box) as classified by Marschalek 2017,

**Figure S3. *In silico* analysis of predicted function of MLL1-fusion partners represented using Cytoscape.** Interactions of MLL1-fusion genes, grouped based on the Gene Ontology. This network is derived from main network shown in Figure 1A.

**Figure S4. Genetic variations in MLL1-fusion partners using cBioPortal.** Tabulated set of pie charts showing alteration frequency of amplification, deletion or mutation in MLL1-fusion partner genes in multiple cancers as derived from cBioPortal. frequencies >10% alone are shown.

**Figure S5. Copy Number Variations in various cancers.** Prevalence of Copy Number Variations (CNVs) and MLL fusion sites showing additions (Amplifications and Gain) and deletions (Shallow and Deep) in Acute Myeloid Leukemia (LAML), Breast invasive carcinoma (BRCA), Liver hepatocellular carcinoma (LIHC) and Skin Cutaneous Melanoma (SKCM).

**Figure S6. Clone validation and characteristics.** (*A*) Histogram showing percentage of V5 expressing HCT116 cells containing Blasticidin selected clones expressing MLL1-ZC3H13 fusion protein, vector control and parental control (HCT116p), following staining with antibody against V5 tag by flowcytometry. The mean values of 2 experiments with standard error bars have been shown and clones with mean values above controls were considered positive, (*B*) Lagging chromosome (Anaphase lag) observed during mitosis in nuclei of HCT116 C7 and C9 clone cells expressing MLL1-ZC3H13 fusion protein stained with Hoechst (Scale bar – 20 μm).

**Figure S7. Cell cycle analysis using propidium iodide.** Representative histogram from three independent replicates of the DNA profile of MLL1-ZC3H13 fusion expressing selected C7, C9 and vector control after growth for various time points (24 h, 48 h, 72 h and 96 h) showing different phases of cell cycle as percentage values (N=3).

**Figure S8. Cell cycle and division characteristics of clones.** (*A*), Fold level change in cell proliferation showing proliferation rates of C7 and C9 compared to vector control (HCT116 VC) using cell counting by trypan blue staining. Mean values of 3 experiments were used for the graph and standard error bars were also calculated, (*B*) Doubling time of the MLL1-ZC3H13 fusion expressing selected C7, C9 and vector control - Tabulated representation of the mean doubling time values (N=3), (*C*) Phospho-histone H3 staining of mitotic cell population in clones expressing MLL1-ZC3H13 fusion protein C7, C9 and vector control at various time intervals following thymidine block for synchronization and subsequent nocodazole release. The cells were stained using AF488 conjugated Rabbit phospho-histone H3 antibody prior to flowcytometry. Time intervals were denoted in differentially colored lines (0 hr – Red; 9 hr – Blue; 12 hr – Green; 15 hr – Orange) in the overlaid histograms of individual clone and control along with representative mitotic population value after the 15 hr.

**Figure S9. Drug inhibition assay.** (*A*) Graphical representation of percentage of cell death after normalization with DMSO treated cells in C7, C9 and vector control at various time points (Day 3, 6, 9 and 12), as assessed by resazurin viability assay following drug treatment using 10nM, 100nM and 1 μM Pinometostat (EPZ-5676). * P < 0.05, 2-tailed Student’s t test (Red * - HCT116 C7 and Black * - HCT116 C9), (*B*) Trend lines of cell cycle phases in C7, C9 and vector control as assessed PI staining following drug treatment at increasing concentrations (100nM, 1μM and 10μM) Pinometostat (EPZ-5676) for various time points (Day 3, 6, 9, 12 and 15), (*C*) Bar graph showing the fold level increase in dead cells following 7AAD staining of C7, C9 and vector control following shRNA silencing using RFP and DOT1L hairpins (Hp). * P < 0.05, 2-tailed Student’s t test (Red * - HCT116 C7 and Black * - HCT116 C9), (*D*) Bar graph showing decrease in mRNA expression of DOT1L in C7, C9 and vector control following shRNA silencing using RFP and DOT1L hairpins (Hp) and determined by qRT-PCR. * P < 0.05, 2-tailed Student’s t test (Black * - HCT116 C9). The error bars in all the subfigures represent the standard error of the mean (SE).

**Figure S10.** **Correlation and complementation analyses of ZC3H13**. (*A*) Correlation scatter plot between ZC3H13 against PTTG1 in various cancers (N-total sample; SC-Spearman Correlation). Abbreviation of each cancer is represented as described in the TCGA portal, (*B*) Graphical representation of complementation studies using yeast with knockouts of PDS, CAN1 and ZC3H13 variants represented as growth measured as absorbance over time.
